# Supplementary material for: Evaluation of primary HPV-based cervical screening among older women: Long-term follow-up of a randomized healthcare policy trial in Sweden
Source: PLoS Med. 2024 Dec 19;21(12):e1004505. doi: 10.1371/journal.pmed.1004505 (PMC11706452; doi:10.1371/journal.pmed.1004505)
Supplement: S1 Protocol — HPV, human papillomavirus. (PDF) [file pmed.1004505.s002.pdf]

Randomized Implementation of Primary HPV Testing in the Organized Screening for Cervical Cancer in Stockholm

Sponsors and Collaborators
Karolinska Institutet (KI)
Region Stockholm

- Center for Cervical Cancer Elimination, Karolinska University Hospital (KUH)
- Regional Cancer Centre Stockholm-Gotland (RCC)

Investigators
K. Miriam Elfström, KUH/RCC
Joakim Dillner, KI/KUH

Study details

Study Type:	Interventional
Study Design:	Allocation: Randomized<br>Intervention Model: Parallel Assignment<br>Masking: None (Open Label)<br>Primary Purpose: Prevention
Ages Eligible for Study:	30-64
Sexes Eligible for Study:	Female
Accepts Healthy Volunteers:	No

Detailed study description

Background

There are approximately 450 cases of cervical cancer in Sweden each year. Since the organized cervical screening program was started in the 1960s, the number of cases has halved. Human papillomavirus (HPV) causes almost all cases of cervical cancer. The development of cancer is preceded by a persistent infection with HPV. HPV types 16 and 18 account for about 70% of all cervical cancer cases. Vaccination against HPV has been recommended in Sweden for girls 12-18 since 2010.

Cervical screening has been based on the cytology where the appearance of cells are examined microscopically. In 2010, liquid based cytology (LBC) was introduced in Stockholm. The advantage with this method as compared to conventional cytology is that the cells are not destroyed, and the sample material can be used for both cytology and HPV analysis.

As HPV-based screening has been shown to have a better sensitivity to detect high-grade cervical lesions and to provide a longer protection after a negative test as compared to cytology, this study aims to examine the implementation of HPV-based screening in the routine, organized screening program of Stockholm-Gotland.

Purpose

The purpose is to evaluate whether implementation of primary human papillomavirus (HPV) screening in the screening program for cervical cancer improves the program in terms of better cancer protection and better cost efficiency.

Study design and methodology

The study was designed to evaluate the test implementation in a well-controlled and step-wise manner following two phases. In the first phase (Phase 1a, women ages 56-60 that participate in cervical screening will be randomized 1:1 to either HPV- (new policy) or cytology-based screening (old policy). This start-up phase is designed to test the logistics and ensure that the new test performs technically in real-life. Furthermore, the first phase will evaluate whether the new policy is acceptable for sample-takers and participating women. In Phase 1b, women ages 35, 49 and 60 will be included and randomized 1:1 according to the procedures in Phase 1a. Finally, in Phase 2, the remaining women ages 30-60 will be randomized 1:1 to HPV- and cytology-based screening. During implementation in 2014, phases 1b and 2 were collapsed.

An evaluation will be conducted annually to compare how many cases of CIN2+ (and separately cancer) are detected in the new policy (HPV-based screening) as compared to the old policy (cytology-based screening). The disadvantages of the two methods, with regards to healthcare costs and number of follow-up examinations will also be evaluated.

Evaluation of the protection against invasive cervical cancer will be performed after the second screening round has been completed approximately 8 years after enrolment for Phase 1b and Phase 2.

Evaluation of the protection against invasive cervical cancer among older women and the effectiveness of HPV and cytology testing as exiting test at the age of 60 will be performed after 10 years after enrolment for Phase 1a.

Women will be invited to screening using the routine invitation letter. In the letter, information on the analysis methods tested in the study will be included. All data on HPV and cytology tests as well as histopathological results will be collected according to clinical practice in the laboratory system of the region.

Data will be exported to the Regional Cancer Center of Stockholm-Gotland (RCC) and Karolinska Institutet for statistical analysis.

Outcome

- Primary
    - Cervical intraepithelial neoplasia grade 2 or worse (CIN2+)
        - The number of women with CIN2+ detected by primary HPV testing will be compared with the number of women with CIN2+ detected by primary cytology.
- Secondary
    - Cost for the two different diagnostic procedures
        - The cost for the new procedure with HPV test in primary screening will be compared to the routine procedure with primary cytology.

- Invasive cervical cancer: The number of women with invasive cervical cancer detected in the two arms.

Specific selection process

- Inclusion criteria
  - Women ages 30-64, invited to routine cervical screening in the Stockholm-Gotland region during the study period.

  Exclusion criteria – no exclusion criteria

Randomization and power calculation
Randomization will be done using the last digit in the person number of the participating women.

We assume a power of 80% and a statistical significance level of 0.05 with a constant rate of CIN2+ incidence in both groups as well as a similar sensitivity in both groups. Given this, we need 269 cases of CIN2+ in each group to be able to show non-inferiority of HPV-testing as compared to cytology.

Recruitment will be limited as follows given logistics. In Phase 1a, about 8,000 women will be recruited per year, in Phase 1b, about 24,000 women will be recruited per year, and in Phase 2 about 120,000 women will be recruited per year. Sufficient power is expected to be achieved 24 months after the start of the pilot implementation.
