# Supplementary figures and images for: Evaluation of primary HPV-based cervical screening among older women: Long-term follow-up of a randomized healthcare policy trial in Sweden
Source: PLoS Med. 2024 Dec 19;21(12):e1004505. doi: 10.1371/journal.pmed.1004505 (PMC11706452; doi:10.1371/journal.pmed.1004505)

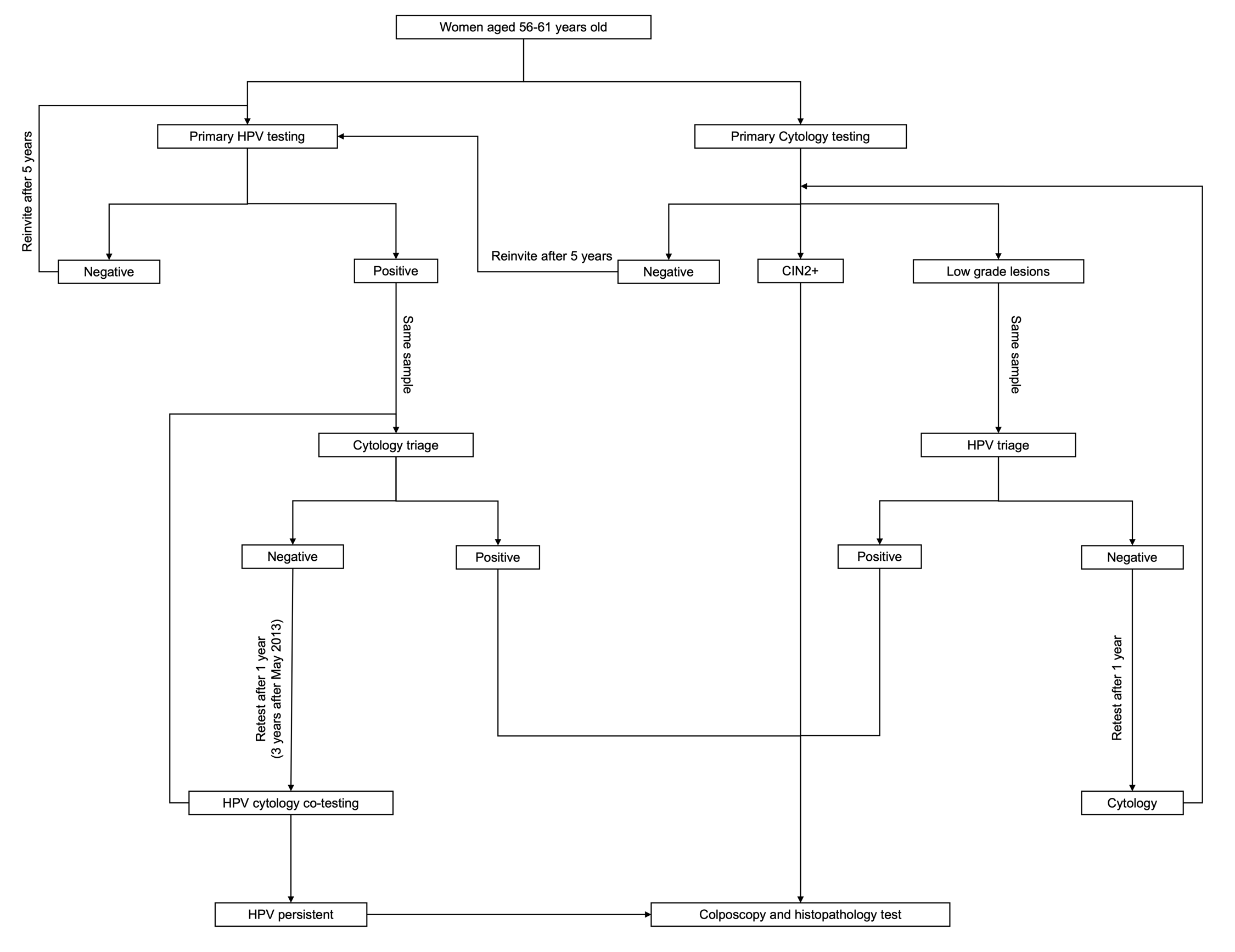

Supplement: S1 Fig — CIN2+, cervical intraepithelial neoplasia grade 2 or worse. (TIFF) [file pmed.1004505.s003.tiff]
