# Supplementary material for: Evaluation of primary HPV-based cervical screening among older women: Long-term follow-up of a randomized healthcare policy trial in Sweden
Source: PLoS Med. 2024 Dec 19;21(12):e1004505. doi: 10.1371/journal.pmed.1004505 (PMC11706452; doi:10.1371/journal.pmed.1004505)
Supplement: S1 Text — (DOCX) [file pmed.1004505.s004.docx]

**Screening protocol**

During the trial, samples were taken by midwives using the same liquid-based cytology system (ThinPrep, Hologic, Boxborough, Massachusetts USA) as used in the routine program. We used the Cobas 4800 HPV Test system (Roche Molecular Systems, South Branchburg, New Jersey, USA) for the HPV analyses. The system tested for HPV 16, 18, 31, 33, 35, 39, 45, 51, 52, 56, 58, 59, 66 and 68 and classified results into the categories HPV 16 positive, HPV 18 positive, other oncogenic HPV positive, or HPV negative. Samples with positive HPV results were subjected to a reflex triage cytology test. During the follow-up, we invited women with a negative result from HPV arm or cytology arm to the next test after 5 years, women with HPV-positive and cytology-negative result were invited to a repeat HPV and cytology test after a year (or after 3 years from May 2013); women with low-grade cytological abnormalities in primary cytology test and negative HPV triage were invited for a repeat test one year later. Women with persistent HPV infection (positive for the same HPV type category), with low-grade cytological abnormalities and HPV positive triage, or with cytology diagnosed CIN2+ (includes CIN2, CIN3, HSIL, ASC-H, adenocarcinoma in situ, AIS, and ICC), were referred to colposcopy. The process flowchart of cervical screening for women in this trial was presented in S1 Fig. The clinical follow-up of both arms was identical. All women referred to colposcopy had routine histopathology tests at the discretion of the colposcopist, both arms following the same guideline for clinical management in Sweden.

**Supplementary statistical analysis**

To investigate the effectiveness of primary HPV test compared to primary cytology test among high-risk women, we also restricted our analysis to women with a previous abnormality and women without a previous abnormality. We categorized women with any previous positive test results, including HPV, cytology, and/or histopathological tests, since the availability of the register data (mostly after 1995) before the randomized trial as women with a previous abnormality. However, due to the low precision caused by a limited number of women with a previous abnormality (n=1208), we were unable to stratify the previous abnormality group based on time elapsed since said abnormality. To investigate whether the risk of histopathology CIN2+ and ICC would increase as a function of previous abnormality, we calculated the IRR comparing women with previous abnormality to women without previous abnormality among all women who participated and stratified by baseline result.

| **Table A**. Number of women who had first organized screening test after the baseline test by baseline sample year and calendar year in each arm. | | | | | | | | | | | | |
| --- | --- | --- | --- | --- | --- | --- | --- | --- | --- | --- | --- | --- |
|  | Cytology Arm | | | | | | HPV Arm | | | | | |
|  | 2012 | | 2013 | | 2014 | | 2012 | | 2013 | | 2014 | |
|  | N | Percent (%) | N | Percent (%) | N | Percent (%) | N | Percent (%) | N | Percent (%) | N | Percent (%) |
| 2012 | 5 | 0.1 | - | - | - | - | 0 | 0 | - | - | - | - |
| 2013 | 32 | 0.4 | 18 | 0.2 | - | - | 8 | 0.1 | 5 | 0.1 | - | - |
| 2014 | 7 | 0.1 | 32 | 0.4 | 7 | 0.1 | 5 | 0 | 5 | 0.1 | 5 | 0.1 |
| 2015 | 1641 | 22.2 | 9 | 0.1 | 8 | 0.1 | 5 | 0.1 | 5 | 0.1 | 5 | 0.1 |
| 2016 | 364 | 4.9 | 1704 | 23.0 | 5 | 0.1 | 7 | 0.1 | 12 | 0.2 | 5 | 0.1 |
| 2017 | 88 | 1.2 | 134 | 1.8 | 5 | 0.1 | 1244 | 17.0 | 21 | 0.3 | 22 | 0.3 |
| 2018 | 28 | 0.4 | 244 | 3.3 | 12 | 0.2 | 190 | 2.6 | 1284 | 17.6 | 7 | 0.1 |
| 2019 | 45 | 0.6 | 138 | 1.9 | 1005 | 13.6 | 78 | 1.1 | 336 | 4.6 | 938 | 12.8 |
| 2020 | 18 | 0.2 | 19 | 0.3 | 100 | 1.4 | 46 | 0.6 | 35 | 0.5 | 114 | 1.6 |
| 2021 | 42 | 0.6 | 89 | 1.2 | 111 | 1.5 | 82 | 1.1 | 178 | 2.4 | 117 | 1.6 |
| 2022 | 165 | 2.2 | 110 | 1.5 | 80 | 1.1 | 539 | 7.4 | 276 | 3.8 | 64 | 0.9 |
| Sum | 2435 | 32.9 | 2497 | 33.7 | 1333 | 18.2 | 2204 | 30.2 | 2157 | 29.7 | 1277 | 17.6 |
| * number equal to or below 5 were all presented as 5; HPV, human papillomavirus. | | | | | | | | | | | | |

| **Table B.** IRR and 95% CI of cervical intraepithelial neoplasia grade 2 or worse (CIN2+) and invasive cervical cancer (ICC) comparing women with primary HPV (human papillomavirus) to primary cytology (reference). | | | | |
| --- | --- | --- | --- | --- |
| Population | CIN2+ | *P* Value | ICC | *P* Value |
| Without a previous abnornality | 1.1 (0.82, 1.6) | 0.4413 | **0.23 (0.07, 0.82)** | **0.0237** |
| With a previous abnormality | 0.91 (0.39, 2.1) | 0.8247 | ∞ | - |
| Women with baseline negative result and no previous abnormality | **0.38 (0.21, 0.66)** | **0.0007** | **0.16 (0.04, 0.72)** | **0.0166** |
| Women with baseline negative result and previous abnormality | 0 | 0.9999 | 0 | 1.0 |
| Women with baseline positive result and no previous abnormality | 1.0 (0.57, 1.7) | 0.9268 | ∞ | - |
| Women with baseline positive result and previous abnormality | 1.0 (0.28, 3.7) | 0.9868 | ∞ | - |
| * CI, confidence interval; IRR, incidence rate ratio. | | | | |

| **Table C.** Adjusted IRR and 95% CI of cervical intraepithelial neoplasia grade 2 or worse (CIN2+) and invasive cervical cancer (ICC) comparing women with primary HPV (human papillomavirus) to primary cytology (reference). | | | | |
| --- | --- | --- | --- | --- |
| Population | CIN2+ | *P* Value | ICC | *P* Value |
| **All population** | 1.1 (0.81, 1.5) | 0.5261 | 0.39 (0.14, 1.1) | 0.0722 |
| Women without previous abnormality | 1.1 (0.81, 1.6) | 0.4492 | **0.23 (0.07, 0.82)** | **0.0233** |
| Women with previous abnormality | 0.94 (0.39, 2.2) | 0.8773 | ∞ | - |
| **Women with negative baseline result** | **0.32 (0.18, 0.55)** | **<0.0001** | **0.16 (0.04, 0.71)** | **0.0161** |
| Women with baseline negative result and no previous abnormality | **0.38 (0.21, 0.66)** | **0.0007** | **0.16 (0.04, 0.72)** | **0.0165** |
| Women with baseline negative result and previous abnormality | 0 | 0.9999 | ∞ | - |
| **Women with positve baseline result** | 1.0 (0.60, 1.6) | 0.9721 | ∞ | - |
| Women with baseline positive result and no previous abnormality | 1.0 (0.58, 1.7) | 0.9692 | ∞ | - |
| Women with baseline positive result and previous abnormality | 1.2 (0.32, 4.4) | 0.7959 | ∞ | - |
| *IRRs were adjusted by baseline sample year; CI, confidence interval; IRR, incidence rate ratio. | | | | |

| **Table D.** IR, IRR and 95% CI of cervical intraepithelial neoplasia grade 2 or worse (CIN2+) and invasive cervical cancer (ICC) of women with previous abnormality compared to women without previous abnormality. | | | | | | | | |
| --- | --- | --- | --- | --- | --- | --- | --- | --- |
|  | **With a previous abnormality** | | | **Without a previous abnormality** | | | **IRR** | ***P* Value** |
|  | n ^*^ | number of cases | IR (/100,000 person-years) | n ^*^ | number of cases | IR (/100,000 person-years) |  |  |
| **CIN2+** |  |  |  |  |  |  |  |  |
| All population | 914 | 22 | 430.2 (283.3, 653.3) | 13505 | 136 | 173.8 (146.9, 205.6) | **2.5 (1.6, 3.9)** | **<0.0001** |
| Cytology | 445 | 12 | 449.6 (255.3, 791.7) | 6816 | 67 | 162.9 (128.2, 207.0) | **2.8 (1.5, 5.1)** | **0.0012** |
| Baseline cytology negative | 432 | 9 | 345.5 (179.8, 664.1) | 6690 | 49 | 121.1 (91.5, 160.2) | **2.9 (1.4, 5.8)** | **0.0038** |
| Baseline cytology positive | 13 | 3 | 4681.9 (1510.0, 14516.8) | 126 | 18 | 2712.9 (1709.2, 4305.9) | 1.7 (0.51, 5.9) | 0.3816 |
| HPV | 469 | 10 | 408.9 (220.0, 760.0) | 6689 | 69 | 185.9 (146.8, 235.4) | **2.2 (1.1, 4.3)** | **0.0198** |
| Baseline HPV negative | 427 | 0 | 0 | 6339 | 16 | 45.6 (27.9, 74.4) | 0 | 0.9998 |
| Baseline HPV positive | 42 | 10 | 4733.2 (2546.7, 8797.1) | 350 | 53 | 2645.8 (2021.3, 3463.2) | 1.8 (0.91, 3.5) | 0.0916 |
| **Invasive Cervical Cancer** |  |  |  |  |  |  |  |  |
| All population | 1208 | 2 | 17.4 (4.4, 69.7) | 13505 | 16 | 12.4 (7.6, 20.2) | 1.4 (0.32, 6.1) | 0.6503 |
| Cytology | 584 | 0 | 0 | 6816 | 13 | 20.0 (11.6, 34.4) | 0 | 0.9997 |
| Baseline cytology negative | 566 | 0 | 0 | 6690 | 13 | 20.3 (11.8, 35.0) | 0 | 0.9997 |
| Baseline cytology positive | 18 | 0 | 0 | 126 | 0 | 0 | ∞ | 1.000 |
| HPV | 624 | 2 | 33.8 (8.4, 135.1) | 6689 | 3 | 4.7 (1.5, 14.5) | **7.2 (1.2, 43.1)** | **0.0305** |
| Baseline HPV negative | 570 | 0 | 0 | 6339 | 2 | 3.3 (0.8, 13.2) | 0 | 0.9999 |
| Baseline HPV positive | 54 | 2 | 407.7 (102.0, 1630.1) | 350 | 1 | 30.3 (4.3, 215.1) | **13.5 (1.2, 148.4)** | **0.0338** |
| *calculation related to CIN2+ exclude women with histopathological diagnosed CIN2+ before the randomized trial; CI, confidence interval; IR, incidence rate; IRR, incidence rate ratio; All population, women participating at baseline; HPV, human papillomavirus. | | | | | | | | |

| **Table E.** Proportion of turning HPV positive, incidence and incidence rate CIN2+ and ICC by re-enter screening program or not among women tested negative at the baseline. | | | | | | | | | |
| --- | --- | --- | --- | --- | --- | --- | --- | --- | --- |
|  |  | Turn HPV positive durimg follow-up | | CIN2+ | | Invasive Cervical Cancer | | |  |
|  | n | number of cases | Percentage (%) | number of cases | IR (/100,000 person-years) | number of cases | IR (/100,000 person-years) | IRR | *P* Value |
| With organized screening test |  |  |  |  |  |  |  |  |  |
| Cytology arm baseline negative | 6166 | 474 | 7.7 | 51 | 122.4 (93.0, 161.0) | 8 | 13.4 (6.7, 26.7) | Ref. |  |
| HPV arm baseline negative | 5423 | 260 | 4.8 | 10 | 28.9 (15.5, 53.7) | 1 | 1.9 (0.3, 13.5) | 0.14 (0.02, 1.1) | 0.0655 |
| Opportunistic and indicated test or no test |  |  |  |  |  |  |  |  |  |
| Cytology arm baseline negative | 1090 | 44 | 4.0 | 8 | 357.8 (178.9, 715.5) | 5 | 52.6 (21.9, 126.5) | Ref. |  |
| HPV arm baseline negative | 1486 | 52 | 3.5 | 7 | 198.8 (94.8, 417.2) | 1 | 7.5 (1.1, 53.2) | 0.14 (0.02, 1.2) | 0.0751 |
| CIN2+, cervical intraepithelial neoplasia grade 2 or worse; CI, confidence interval; IR, incidence rate; IRR, incidence rate ratio; ref, reference; HPV, human papillomavirus. | | | | | | | | | |
