# Supplementary material for: Evaluation of primary HPV-based cervical screening among older women: Long-term follow-up of a randomized healthcare policy trial in Sweden
Source: PLoS Med. 2024 Dec 19;21(12):e1004505. doi: 10.1371/journal.pmed.1004505 (PMC11706452; doi:10.1371/journal.pmed.1004505)
Supplement: S1 Statistical Analysis Plan — (PDF) [file pmed.1004505.s005.pdf]

Analysis_plan_**project- Effectiveness of primary HPV-based cervical screening among older women: Long-term follow-up of a randomised healthcare policy trial**.rtf/.docx
Created: 2023-09-12 by Qingyun Yao

1 OBJECTIVE AND HYPOTHESES

The objective of this study is to compare the effectiveness of primary HPV test versus cytology screening among post-menopausal women. Using cohort study to examine the incidence rate of CIN2+ among women aged 65-70 years. comparing those who at age 56-61 had negative primary HPV tests to those who had only cytology tests with normal results. In addition. we will compare the effectiveness of HPV tests and cytological tests among those who had previous positive screening results. to explore whether a negative HPV screening test regardless of previous screening outcomes would provide enough protection for 7 years among women aged 56-61.In order to provides evidence for the exiting screening test and to adjust the screening policy among postmenopausal women.

Hypothesis

1. Women with a negative primary HPV test have lower chance of developing a CIN2+ lesion than women with a negative cytological test.
2. A negative primary HPV test can provide protection against invasive cervical cancer for women aged 56-61 years old for 7 years.
3. Among women with any previous positive result. Women with a negative primary HPV test have lower chance of developing a CIN2+ lesion than women with a negative cytological test.
4. Among women with any previous positive result. one negative HPV test can provide enough protection for 7 years against invasive cervical cancer.
5. Women with previous abnormality will have higher chance of positive result is the following screening, even current screening result is negative (only among women with fas1 negative result)

2 NOTATION AND ABBREVATIONS

CIN system

With cervical intraepithelial neoplasia (CIN). abnormal cells solely confined to the lower third if the squamous epithelium are referred to as mild dysplasia or CIN1. Those that extend into the middle third are moderate dysplasia or CIN2; into the upper third. severe dysplasia or CIN3; and full-thickness involvement. carcinoma in situ (CIS). (Hoffman. B. L. et al. 2020)

Bethesda system (Solomon D. et al. 2002)

HSIL: High-grade squamous intraepithelial lesion (HSIL or HGSIL) indicates moderate or severe cervical intraepithelial neoplasia or carcinoma in situ. HSIL generally corresponds to the histological classification of CIN 2 or 3

LSIL: A low-grade squamous intraepithelial lesion (LSIL or LGSIL) indicates possible cervical dysplasia. LSIL usually indicates mild dysplasia (CIN 1).

Terminology standardized based on the Darragh TM. et al. (Darragh TM. et al. 2013)

Cytological test SNOMED severity in NKCx:

5----Koilocytosis 6----ASCUS; 7----CIN1 LSIL; 8----AGC LSIL; 9----unclear atypia LSIL; 10---suspected high grade dysplasia ASC-H; 11---CIN2 HSIL; 12---CIN3 HSIL; 13---invasive adenocarcinoma HSIL; 14---Malignant tumor with unclear origin HSIL

PAD SNOMED severity in NKCx:

1---benign; 3---ASCUS; 4---CIN1 LSIL; 5---CIN2 HSIL; 6---CIN3 HSIL; 9---cancer HSIL

The definition of cytological abnormality in this cohort is the cytological SNOMED severity in NKCx database >=6

The definition of cytological diagnosed LSIL in this cohort is the cytological SNOMED severity in NKCx database >=7

The definition of cytological diagnosed HSIL in this cohort is the cytological SNOMED severity in NKCx database >=11

The definition of cytological diagnosed cancer in this cohort is the cytological SNOMED severity in NKCx database >=13

The definition of pathological diagnosed LSIL in this cohort is the pad severity in NKCx database >=4

The definition of pathological diagnosed HSIL in this cohort is the pad severity in NKCx database >=5

The definition of pathological diagnosed cancer in this cohort is the pad severity in NKCx database >=9

3 STUDY POPULATION

During Jan 2012 to May 2014. women aged 56 to 61 years old (age defined by birth year. in this study women were born between 1 Jan 1951 and 31 Dec 1958). resident in the Stockholm-Gotland region of Sweden were invited to their last screening test based on the cervical cancer screening policy before 2017 (Hortlund M. et al. 2018). They were randomized into two groups: (1) primary cytological test with triage HPV test for women with low grade cytological abnormality (ASCUS or LSIL); and (2) primary HPV test with triage cytological test for women with positive HPV results. The randomization is based on the last digit number of personal identity number in Sweden. (Women invited n=14719). (Lamin H. et al. 2016). This randomized trial will be referred to as Fas1 in the following of this document.

3.1 INCLUSION CRITERIA

Have a screening test result at Fas1 trial.

There are 6909 women underwent primary HPV test with a negative result and 7256 women underwent primary cytological test with a negative result.

3.2 EXCLUSION CRITERIA

Women who have hysterectomy/personal choice (The hysterectomy is identified in NKC_deregister variable X_DEREG_REASON value: Hysterektomi/egen vilja) before Fas1 trial were excluded (n=3 person)

4 MEASUREMENTS AND VARIABLES

4.1 Outcome - efficacy variables

Data resource:

NKCx: All tests related to HPV and cytology screening (including all cervical biopsies) in Sweden are recorded in the Swedish National Cervical Screening Registry (Swedish acronym NKCx). Screening was initiated in 1970s and the registry reached full coverage nationally since 1995. with around 800.000 tests recorded annually.

Gynecological Cancer Quality Registry: From 2011. detailed information of cervical cancer diagnoses in whole Sweden was also recorded in the Swedish National Quality Register for Gynecological Cancers (QGCR). (Rosenberg P. et al 2017) (the coverage for the ovarian cancer and endometrial cancer, which is 94% and 96% respectively)

National Cancer Registry (NCR): The NCR was founded in 1958 and now has a high level of validity and full coverage of malignant cancer in Sweden. (Barlow L. et al 2009)

These above mentioned registries are all linkable on individual level with very high accuracy through a personal identification number (Ludvigsson JF. et al 2016)

Variable name in the data set: p_HSIL
Description: Follow up ending. Pathological diagnosed HSIL or worse abnormality

Format: number

Value description: 0-no;1-yes

Variable name in the data set: Cancer

Description: pathological diagnosed cancer. The data is from GCQR

Format: number

Values description: 0-no.1-yes

Variable name in the data set: HSIL_survival_time

Description: Time interval between the fas1 test date and the end of follow up of HSIL. End of follow up of ending HSIL and worse abnormality: for women without abnormality. it will be the date of the last test; for women with an abnormality. it will be the date of diagnosis.

Format: num unit: years

Variable name in the data set: Can_survival_time

Description: Time interval between fas1 test date and either the diagnosis date of cancer or the nearest recorded date in the GCQR registry (2022/12/31)

Format: num unit: years

***

4.2 Exposure covariates

Variable name in the data set: group

Description: variable set up for proc phreg; based on the fas1 primary test results

Format: chr
Values description: Cyt_N-cytological negative; Hpv_N-hpv negative; CYT_P cytological positive; Hpv_P hpv positive

Variable name in the data set: HPV1618

Description: HPV genotype information. based on data set nkc_extended_hpv (This data set start in 2013. Doesn't have hpv type information for test in 2012). differentiate hpv 16/18 and other hpv type

Format: num

Value description: 2-hpv16/18 positive; 1-Other hpv type positive; 0- hpv negative

***

4.3 Mandatory covariates. known confounders

Fas1_sample_yr: the year the participant joined the fas1 trial

Reasons for including these covariates*

There is an imbalance in invitation between primary cytology and primary HPV group. The imbalance only exist among women had fas1 test in year 2012 and year 2013. We adjusted sample year in poisson regression to calculated the adjusted incidence rate ratio

4.4 Additional covariates. potential confounders

None

Reasons for including these covariates*

Our study is based on a randomized trial with no detection of imbalances. therefore there is no need to adjust for additional covariates.

4.5 Effect Modification

none

Reasons for including these covariates*

5 DATA MANAGEMENT

*** raw data files. program files etc. and documentation files ***
The files in this project are stored in P:\ACCES\ACCES_Research\Qingyun\Fas1_Followup
The logbook (LOGBOOK_fas1_followup_QY.doc) can be found in Documents folder.

6 STATISTICAL ANALYSES

***

- Kaplan-Meier curves to estimate CIN2+ and ICC survival probability
- Longitudinal characteristics (sensitivity and specificity) of HPV and cytology to identify CIN2+ lesions at 3, 5, 7, and 10 years were calculated with conditional weighting
- Incidence rate by primary test result and pre-abnormality
- Incidence rate ratios, primary cytology compared to primary HPV, adjusted IRR using poisson regression (adjusted for sample_year)
- Incidence rate ratios, women with pre-abnormality compared to women without pre-abnormality
- Cox regression modeling for the hazard ratio HSIL+ and ICC

7 STAFF LIST

Contact information to researchers and staff involved in the study:

Karin Sundström. PI. last author. karin.sundstrom@ki.se. CLINTEC
Qingyun Yao. PhD student. first author qingyun.yao@ki.se
Jiangrong Wang. Post-doc. jiangrong.wang@ki.se
Miriam Elfström, PhD, miriam.elfström@ki.se
Björn strander, professor, MD, PhD, bjorn.strander@gu.se
Joakim Dillner, professor, MD, PhD, joakim.dillner@ki.se

8 REFERENCES

Darragh. Teresa M.. Terence J. Colgan. J. Thomas Cox. Debra S. Heller. Michael R. Henry. Ronald D. Luff. Timothy McCalmont. et al. "The Lower Anogenital Squamous Terminology Standardization Project for HPV-Associated Lesions: Background and Consensus Recommendations From the College of American Pathologists and the American Society for Colposcopy and Cervical Pathology." *International Journal of Gynecological Pathology* 32. no. 1 (January 2013): 76–115. https://doi.org/10.1097/PGP.0b013e31826916c7.

Inturrisi F. Rozendaal L. Veldhuijzen NJ. Heideman DAM. Meijer CJLM. Berkhof J (2022) Risk of cervical precancer among HPV–negative women in the Netherlands and its association with previous HPV and cytology results: A follow-up analysis of a randomized screening study. PloS Med 19(10): e1004115. https://doi.org/10.1371/journal.pmed.1004115

Hoffman. B. L.. MD (2020). *Williams Gynecology* (4th ed.. p. 620). Mc Graw Hill.

Solomon D. Davey D. Kurman R. et al. The 2001 Bethesda System: Terminology for Reporting Results of Cervical Cytology. *JAMA.* 2002;287(16):2114–2119. doi:10.1001/jama.287.16.2114

Hortlund. Maria. K. Miriam Elfström. Pär Sparén. Pouran Almstedt. Björn Strander. and Joakim Dillner. "Cervical Cancer Screening in Sweden 2014-2016." *PLoS ONE* 13. no. 12 (December 17. 2018): e0209003. https://doi.org/10.1371/journal.pone.0209003.

Lamin H. Eklund C. Elfström KM. Carlsten-Thor A. Hortlund M. Elfgren K. Törnberg S. Dillner J. Randomised healthcare policy evaluation of organised primary human papillomavirus screening of women aged 56-60. BMJ Open. 2017 May 30;7(5):e014788. doi: 10.1136/bmjopen-2016-014788. PMID: 28566363; PMCID: PMC5729984.

Swedish National Cervical Screening Registry_Analysis. NKCx; 2022. Available from: https://nkcx.se/index_e.htm

Svenska kvalitetsregistret för gyologisk cancer. Available from: https://cancercentrum.se/samverkan/cancerdiagnoser/gynekologi/kvalitetsregister/

National cancer register. Available from: https://www.socialstyrelsen.se/en/statistics-and-data/registers/national-cancer-register/

Ludvigsson JF. et al. Registers of the Swedish total population and their use in medical research. Eur J Epidemiol 2016;2: 125-136.

Meinert. C. L.. G. L. Knatterud. T. E. Prout. and C. R. Klimt. "A Study of the Effects of Hypoglycemic Agents on Vascular Complications in Patients with Adult-Onset Diabetes. II. Mortality Results." *Diabetes* 19 (1970): Suppl:789-830.

Rosenberg. Per. Preben Kjølhede. Christian Staf. Maria Bjurberg. Christer Borgfeldt. Pernilla Dahm-Kähler. Kristina Hellman. et al. "Data Quality in the Swedish Quality Register of Gynecologic Cancer – a Swedish Gynecologic Cancer Group (SweGCG) Study." *Acta Oncologica* 57. no. 3 (March 4. 2018): 346–53. https://doi.org/10.1080/0284186X.2017.1366048.

Barlow. Lotti. Kerstin Westergren. Lars Holmberg. and Mats Talbäck. "The Completeness of the Swedish Cancer Register: A Sample Survey for Year 1998." *Acta Oncologica (Stockholm. Sweden)* 48. no. 1 (2009): 27–33. https://doi.org/10.1080/02841860802247664.

9 ETHICAL APPROVALS

DNR 2011/1298-31/3
