# Supplementary material for: Evaluation of primary HPV-based cervical screening among older women: Long-term follow-up of a randomized healthcare policy trial in Sweden
Source: PLoS Med. 2024 Dec 19;21(12):e1004505. doi: 10.1371/journal.pmed.1004505 (PMC11706452; doi:10.1371/journal.pmed.1004505)
Supplement: Attachment — Submitted filename: PLOS2024.docx [file pmed.1004505.s006.docx]

Primary HPV testing had a higher sensitivity of detecting CIN2+ within a 7-year interval than primary cytology test within a 5-year interval (89.6% vs 50.9%).

This study also showed that older women with an HPV negative result had higher risk of subsequent CIN2+ if they had a previous cytology positive test result. These results indicate that leaving with a cytology negative test at the age of 60 in general is not safe, given the high incidence rate of ICC after 10 years, at least in women with previous abnormalities

1. Only 35% of countries worldwide 78 currently endorse HPV-based screening in their national recommendations. The recommended criterion of exiting program differs between women with only negative screening history within the past 10-year period, or women with one negative HPV test when arriving at the upper age limit. What is the case in most European countries? Can you please provide these data?
2. There is also a lack of evidence of when it is appropriate to exit the screening. Most countries recommend women exit screening around 60-70 years old. This study supports the extending of age upper limit for cervical screening. Women with their last organized screening test above the age of 55 still have a risk of invasive cervical cancer higher than the elimination threshold. Till what age would the authors suggest screening? Would this be HPV-based, cytology based? Or a combination?
3. This is one the first study that compared the longitudinal characteristics of HPV and cytology in detecting CIN2+ or ICC among older women by different time intervals. Table 3 shows longitudinal test characteristics using cervical intraepithelial neoplasia grade 2 or worse (CIN2+) as outcome at 3, 5, 7 and 10 years of followup. What would be the guideline interval? Based on what? Would this be different between women with previous abnormal smears?
4. During the follow-up, women were invited women with a negative result from HPV arm or cytology arm to the next test after 5 years, women with HPV positive and cytology-negative result were invited to a repeat HPV and cytology test after a year (or after 3 years from May 2013); women with low-grade cytological abnormalities in primary cytology test and negative HPV triage were invited for a repeat test one year later. Women with persistent HPV infection (positive for the same HPV type category), with lowgrade cytological abnormalities and HPV positive triage, or with CIN2+, were referred to colposcopy. Could you please pout these data in a process flowchart?
