# Supplementary material for: Evaluation of primary HPV-based cervical screening among older women: Long-term follow-up of a randomized healthcare policy trial in Sweden
Source: PLoS Med. 2024 Dec 19;21(12):e1004505. doi: 10.1371/journal.pmed.1004505 (PMC11706452; doi:10.1371/journal.pmed.1004505)
Supplement: Attachment — Submitted filename: response to reviewer_QY.docx [file pmed.1004505.s007.docx]

**Response to Reviewers: Effectiveness of primary HPV-based cervical screening among older women: Long-term follow-up of a randomised healthcare policy trial**

Dear Editors,

Many thanks for giving us the opportunity to resubmit a revised manuscript to PLOS Medicine for your consideration. We have carefully revised out paper accoding to the reviewers and editors’ comment. The detailed responses are liste below, point by point.

***Please note that all the line numbers and page numbers cited below are as appear in the fully expanded track change version of the revised manuscript.***

**Reviewer #1:** I have not identified any issues that need to be addressed.

**Response:** We thank the reviewer for the supportive statement.

**Reviewer #2:** Thanks for the opportunity to review the paper entitled "Effectiveness of primary HPV-based cervical screening among older women: Long-term follow-up of a randomized healthcare policy trial on exit testing"

The paper describes risk of CIN2+ and cervical cancer in older women who underwent cytology vs HPV-based screening as part of a clinical trial. The authors conclude that an HPV-negative test provides greater re-assurance against cervical cancer than a negative cytology.

I find that the paper is important and interesting. Results are clinically relevant. Overall, the paper is well written but there are typos and missing words occasionally (P 6, l 112;  P25, l 326; p26, line 363, etc), and some phrases are hard to understand. Please find below my detailed comments, which I hope will be useful for the authors.

**Response:** Thank you for considering our work as important and interesting. According to your suggestions, we have carefully revised our manuscript and corrected typos and missing words. The detailed responses are listed below, point by point.

**Revised text in Introduction:**

**Page 8 lines 240-242** We also compared the effectiveness of primary HPV and primary cytology test among women with or without previous abnormal screening history, respectively.

**Revised text in Discussion:**

**Page 24 lines 588-590** This difference may be explained by the larger size of our trial in this age group and by factors such as our program using a different HPV-testing platform, the more sensitive cobas 4800, as compared to the Hybrid Capture 2 method used in the Finnish study [26–28].

**Revised text in Discussion:**

**Page 26 lines 674-677** However, evidence from both our study and the POBASCAM trial indicates that women with previous abnormal screening history have a higher risk of precancer lesion during long-term follow-up [32]. Risk stratification based on previous screening history may be motivated.

**Comment 1.**      Title: Consider deleting "on exit testing".

**Response:** Thanks for the comment, we have revised the title accordingly.

**Revised text in Title:**

**Page 1 lines 1-2** Effectiveness of primary HPV-based cervical screening among older women: Long-term follow-up of a randomised healthcare policy trial

**Comment 2.**      Abstract: a.  second sentence in the background is long and hard to understand. Please revise/split up.

**Response:** Thanks for the comment, we have revised the sentence accordingly.

**Revised text in Abstract:**

**Page 3 lines 40-44** We conducted a long-term follow-up of the first phase of a randomized healthcare policy trial in cervical screening, targeting women aged 56-61 years old, to investigate the effectiveness of primary HPV-based screening in preventing invasive cervical cancer (ICC) and the safety of extending screening interval.

**Comment 3.**      Abstract: b.      Result headline is missing.

**Response:** Thanks for the comment. According to the journal’s formatting requirments, the abstract is constructed with only Background, Methods and Finding, and Conclusion. We include our results in the second section.

**Comment 4.**      Abstract:c.      Please revise the following. Either delete an 84% or delete statistically significant : "Among women with a negative test result at baseline, women in THE primary HPV arm had an 84% statistically significant lower risk of ICC compared to women in the primary cytology arm (IRR: 0.16; 95% CI: 0.04, 0.72). "

**Response:** Thanks for the comment. We have revised accordingly.

**Revised text in Abstract:**

**Page 4 lines 73-75** Among women with a negative test result at baseline, women in primary HPV arm had an 84% lower risk of ICC compared to women in the primary cytology arm (IRR: 0.16; 95% CI: 0.04, 0.72, *p*=0.0163).

**Comment 5.**           Introduction: a.      P5, lines 75-76: another (and very important) reason is the diagnostic difficulties in this group of women. This is often an overlooked issue and should be stated here.

**Response:** Thanks for the comment. The diagnostic difficulties caused by cervical atrophy changes in this group of women leads to lower sensitivity of cytology and colposcopy. In addition, using low sensitivity tests in screening among older women is considered as a suboptimal screening program design in our opinion. To be more clear we have revised the text as follows.

**Revised text in Introduction:**

**Page 6 lines 116-119** The relatively higher incidence of cervical cancer and cervical intraepithelial neoplasia grade 2 or worse (CIN2+) among older women has been associated with suboptimal screening participation, diagnostic difficulties due to morphological changes due to mucosal atrophy due to menopause, and controversy in screening upper age limit [1,4].

**Comment 6.**           Introduction: b.      P5, line 81: I guess it differs between countries and not women?

**Response:** Thanks for the comment.We have revised accordingly.

**Revised text in Introduction:**

**Page 6 lines 127-132** The recommended criterion of exiting the program differs between countries. Based on recommendations from American Cancer Society, only women without a history of CIN2+ within past 25 years and with adequate negative screening history within the past 10-year period can discontinue screening [8]. The European guidelines suggest women with one negative HPV test when arriving at the upper age limit (60 or 65 years) can discontinue screening [9].

**Comment 7.**           Introduction: c.      P6, line 96. Delete previously (redundant)

**Response:** Thanks for the comment.We have revised accordingly.

**Revised text in Introduction:**

**Page 7 line 204** Several studies have subsequently confirmed these results [16,17].

**Comment 8.**           Introduction: d.      P6, line 101. This sentence is unclear to me. Please elaborate. This seems to be important information to the reader, and readers should not have to read another paper to understand.

**Response:** Thanks for the comment. At the time of the trial initiated, the Swedish guideline for cervical screening recommended to screen women aged over 50 years old with a 5-year interval. The upper limit age for screening was 60 years old. Women would thus receive their last screening test at the age around 56-61 years old. Based on the guideline at that time, we implemented a randomized policy trial in 2012 to compare the effectiveness of primary HPV testing and primary cytology testing as exiting test. In 2017, the updated guideline with HPV-based screening was implemented in Stockholm. The new guideline recommends to screen women aged over 50 years old with a 7-year interval and the upper limit age for screening was postponed to 64 years old. All women have been screened at least once under the new guideline ever since. We have revised our introduction to make the background of this randomized policy trial more clear.

**Revised text in Introduction:**

**Pages 7-8 lines 190-233** In 2012, we began implementing a 2-phase randomized healthcare policy trial inviting all eligible women aged 30-64 residing in the capital Stockholm-Gotland region. The first phase, reported here, targeted women aged 56-61 during 2012-2014 and the second phase targeted women aged above 30 years. The goal of both phases, respectively, was to investigate the detection rate of CIN2+ within 24 months and long-term protection against ICC, comparing primary HPV screening to primary cytology screening. At the time of the trial initiation, the Swedish guidelines for cervical screening recommended to screen women aged over 50 years with primary cytology and a 5-year interval. The upper limit age for screening at that time was set to 60 years old, which resulted in women receiving the exit testing at the age around 56-61 years. Based on this, the first phase of the randomized healthcare policy trial aimed to compare the effectiveness of primary HPV testing and primary cytology testing as exiting test in this age group [14,15]. We have previously reported similar baseline detection rates of CIN2+ whether using primary HPV testing or primary cytology among older women in a non-inferiority randomized trial of exit testing with HPV compared to exit testing with cytology, in 2012 in Sweden [14]. Several studies have subsequently confirmed these results [16,17].

In 2017, updated guidelines recommending primary HPV-based screening were implemented in Stockholm, where women aged over 50 years started receiving primary HPV test screening with an 7-year interval and with the upper age limit set at 64 years. The guidelines were also clarified to state that all women should receive HPV testing rather than cytology at exit from screening.

**Comment 9.**           Methods: a.      P7, lines 115-117. This is unclear. Please specify.

**Response:** Thanks for the comment. We have elaborated the background and purpose of this randomized healthcare policy trial in response to comment 8. We have revised our introduction to make the background of this randomized policy trial more clear.

**Comment 10.**           Methods: b.      P7, line 124: CIN1 is a histologic diagnosis and should not be used for cytological grading. Please delete.

**Response:** Thanks for the comment. We understand that CIN1 in many settings is a histopathological diagnosis. However, it was used in Sweden for a long time as also a cytological diagnosis, use which is supported by the reference [1]. From 2017, all the cytology diagnosis system in Sweden has changed to LSIL and HSIL. However the historical cytology diagnosis still has CIN1, CIN2, CIN3 etc. This we cannot change. We have revised the manuscript and now specify when referring to cytology diagnosed CIN1 and CIN2 etc.

Reference:

1. “Colposcopy and Treatment of Cervical Intraepithelial Neoplasia: A Beginners’ Manual.” Accessed August 13, 2024. <https://screening.iarc.fr/colpochap.php?lang=1&chap=2>.

**Comment 11.**           Methods: c.      P8, line 140: CIN2+?? How can women have a histological diagnosis of CIN2+. Do you mean high-grade cytology? (HSIL, AIS, AGC, ASC-H)?

**Response:** Thanks for the comment. The motivation of using CIN2+ is mentioned in the response to comment 10. We have revised to specify if the CIN2+ is a cytology diagnosis. The cytology diagnosed CIN2+ includes CIN2, CIN3, ASC-H, adenocarcinoma in situ, AIS, and ICC. We have also specified this in our supporting information.

**Revised text in Supporting information (S4 Text):**

**Supporting information S4 Page 1 lines 13-16** Women with persistent HPV infection (positive for the same HPV type category), with low-grade cytological abnormalities and HPV positive triage, or with cytology diagnosed CIN2+ (includes CIN2, CIN3, HSIL, ASC-H, adenocarcinoma in situ, AIS, and ICC), were referred to colposcopy.

**Comment 12.**           Methods: d.      P8, lines 142: Please elaborate. As only women who have a biopsy collected are at risk of CIN2+ and ICC, it is important to know if all women have one or multiple biopsies collected or if biopsies are collected at the discretion of the colposcopist. Also, it is important to know if LLETZ were part of the diagnostic work-up as well or if you only collected biopsy results. A previous study on women in the same age group has revealed that biopsies (when collected) miss 54% of CIN2+ cases.

**Response:** Thanks for the comment. According to the guideline for clinical management in Sweden at the time, women referred to colposcopy would normally have a routine histopathology test, but the decision ultimately resided at the discretion of the colposcopist. In the registry we only have information on histopathology test and diagnosis made. It is not known from the registry data if said diagnosis originated from a cervical biopsy or eg. a LLETZ sample. To avoid bias, we describe all of them simply as histopathology tests.

**Revised text in Method:**

**Page 10 lines 306-310** We used the Swedish National Cervical Screening Registry (NKCx), which has a full coverage nationally of cervical cancer screening results since 1995 [20], including all organized screening program tests, all non-organized/opportunistic tests, all indicated tests due to e.g. clinical symptoms, and all cervical histopathology tests and results, to retrieve all the screening tests and test results from the trial and subsequently during our follow-up.

**Revised text in Supporting information (S4 Text):**

**Supporting information S4 Page 1 lines 17-19** The clinical follow-up of both arms was identical. All women referred to colposcopy had routine histopathology tests at the discretion of the colposcopist, both arms following the same guideline for clinical management in Sweden.

**Comment 13.**           Methods: e.      P8, line 159. Please continue to use the Bethesda classification. Thus, replace koilocytosis with ASCUS+

**Response:** Thanks for the comment. Revised accordingly.

**Revised text in Method:**

**Page 10 lines 315-316** We categorized the baseline test results as HPV positive (regardless of HPV type), HPV negative, cytology positive (ASCUS+), and cytology negative.

**Comment 14.**           Methods: f.      P8, line 161-p9, 169. This section is confusion and needs to be rephrased. I assume you are referring to the two analyses; CIN2+ and ICC. Follow-up did not start after their diagnosis, right? But rather women contributed time at-risk from baseline until a record of CIN2+ and ICC, respectively. You only state censoring events for ICC, but I assume the same censoring events are applicable to the CIN2+ analysis (death, emigration, hysterectomy). Also, women who undergo hysterectomy due to benign disease should be censored and not just the ones that had a hysterectomy due to cancer. Also, please specific what CIN2+ includes (I assume, CIN2, CIN3, AIS, and all cancers).

**Response:** Thanks for the comment. The follow-up started right after the first sample taken during 2012-2014 as reported in the Method section (page 10, lines 314-315). The NKCx database includes all tests related to cervical cancer, whether it is an organized screening program-derived test, opportunistic test, or symptom related indicated tests in the clinic. It also contains all the histopathology results related to the cervix. In the analysis of histopathology diagnosed CIN2+, the follow-up time is defined as the time elapsed from baseline to either the first histopathological diagnosis of CIN2+, or the date of the last registered test, including cytology, HPV, and histopathology before 31^st^ Dec 2022. We have revised our manuscript to make this clearer. As long as there was a test registered, we assume the women was still alive and live in Sweden.

In the analyses of ICC as outcome, we censored all women at the date of total hysterectomy regardless of the cause, which means we censored both women who had hysterectomy due to dysplasia or cancer and women who had hysterectomy due to benign disease. Histopathology diagnosed CIN2+ included CIN2, CIN3, AIS (adenocarcinoma in situ) and invasive cervical cancer, as per international standards. We have revised the description of the database to make the data sources more clear, specified what CIN2+ includes and also what types of hysterectomy was included in our manuscript.

**Revised text in Method:**

**Page 10 lines 306-310** We used the Swedish National Cervical Screening Registry (NKCx), which has a full coverage nationally of cervical cancer screening results since 1995 [20], including all organized screening program tests, all non-organized/opportunistic tests, all indicated tests due to e.g. clinical symptoms, and all cervical histopathology tests and results, to retrieve all the screening tests and test results from the trial and subsequently during our follow-up.

**Page 10 lines 316-321** We identified CIN2+ (includes CIN2, CIN3, AIS, ICC) diagnosed by a histopathology test in the NKCx. In the analysis of histopathology-confirmed CIN2+ as outcome, the follow-up time was defined as the time elapsed from the baseline test to either the first histopathological diagnosis of CIN2+, or the date of the last registered test, including cytology, HPV, and histopathology before 31^st^ Dec 2022.

**Page 10 lines 326-328** End of ICC follow-up was defined as the first cancer diagnosis in the QGCR, the date of emigration, death, or total hysterectomy in the NKCx, or 31^st^ Dec 2022, whichever came first.

**Comment 15.**           Methods: g.      It would be helpful for the reader if you could state the primary and secondary outcome

**Response:** Thanks for the comment. The primary outcome was the incidence of invasive cervical cancer and the secondary outcome was the incidence of histopathology CIN2+ lesions. We have added this in our Methods.

**Revised text in Method:**

**Page 11 lines 346-347** Our primary outcome was the incidence of ICC, and our secondary outcome was the incidence of histopathology-confirmed CIN2+ lesions.

**Comment 16.**           Methods: h.      I would be helpful to know more about previous screening history. How was this information collected and defined? Any abnormal cytology or histology only??

**Response:** Thanks for the comment. The NKCx database includes all tests related to cervical cancer, whether it is an organized screening program-derived test, opportunistic test, or symptom related indicated tests in the clinic. It also contains all the histopathology results related to the cervix. The NKCx started to have complete coverage of the whole nation, including the whole Stockholm region in 1995. The variable “previous abnormality” is based on availability of the registry data. The definition is therefore indeed any positive test results ever in NKCx before the trial baseline test, including cytology (ASCUS+), HPV positive, and/or histologically confirmed CIN1+. We have revised the text to clarify.

**Revised text in Supporting information (S4 Text):**

**Supporting information S4 Page 1 lines 25-28** We categorized women with any previous positive test results, including HPV, cytology, and/or histopathological tests, since the availability of the register data (mostly after 1995) before the randomized trial as women with a previous abnormality.

**Comment 17.**           Methods: i.      Statistics is very long. Suggest condensing.

**Response:** Thanks for the comment. To improve the readibility of the manuscript, we moved the work flow of screening in this trial and the analysis concerning previous abnormality into supporting information (S4 Text).

**Comment 18.**           Methods: j.      Why exclude women with a previous history of CIN2+

**Response:** Thanks for the comment. Women with a previous CIN2+ histopathology history may have different behavior and different attitude towards screening. Therefore we limited our population among women without previous histopathology CIN2+ history in analysis of histopathology-confirmed CIN2+. We tried to evaluate the effectiveness of primary HPV-based screening in identifying the first occurence of histopathology CIN2+ and ICC in our study. We have revised our manuscript to make this more clear. We performed a sensitive analysis without excluding women with previous histopathology-confirmed CIN2+ (Table R1-R3). The results remained unchanged.

**Revised text in Method:**

**Pages 12-13 lines 412-426** To calculate the incidence of the first occurrence of histopathology-confirmed CIN2+, all analyses using histopathology-confirmed CIN2+ as an outcome excluded women with histopathological diagnosed CIN2+ before the recruitment (n=294) except in the subgroup analysis.

| **Table R1.** Incidence rate, incidence rate ratio, and 95% CI of cervical intraepithelial neoplasia grade 2 or worse (CIN2+) | | | | | |
| --- | --- | --- | --- | --- | --- |
|  | CIN2+ | | | | |
|  | n ^*^ | number of cases | IR (/100,000 person-years) | IRR | *P* value |
| All population | 14713 | 166 | 195.2 (167.6, 227.2) |  |  |
| Cytology | 7400 | 81 | 181.3 (145.9, 225.5) | Ref. |  |
| HPV | 7313 | 85 | 210.4 (170.1, 260.3) | 1.2 (0.86, 1.6) | 0.3383 |
| Women with negative baseline result | | | | |  |
| Cytology | 7256 | 59 | 134.4 (104.1, 173.4) | Ref. |  |
| HPV | 6909 | 17 | 44.6 (27.7, 71.7) | **0.33 (0.19, 0.57)** | **<0.0001** |
| Women with positive baseline result | | | | |  |
| Cytology | 144 | 22 | 2888.7 (1902.1, 4387.2) | Ref. |  |
| HPV | 404 | 68 | 2995.8 (2362.0, 3799.6) | 1.0 (0.64, 1.7) | 0.8820 |
| CI, confidence interval; IR, incidence rate; IRR, incidence rate ratio; ref, reference; all population, women participating at baseline; | | | | | |

| **Table R2.** IR, IRR and 95% CI of cervical intraepithelial neoplasia grade 2 or worse (CIN2+) and invasive cervical cancer (ICC) of women with previous abnormality compared to women without previous abnormality after excluding women without any previous screening history | | | | | | | | |
| --- | --- | --- | --- | --- | --- | --- | --- | --- |
|  | **With a previous abnormality** | | | **Without a previous abnormality** | | | **IRR** | ***P* Value** |
|  | n | number of cases | IR (/100,000 person-years) | n | number of cases | IR (/100,000 person-years) |  |  |
| **CIN2+** |  |  |  |  |  |  |  |  |
| All population | 1208 | 30 | 439.4 (307.2, 628.4) | 13505 | 136 | 173.8 (146.9, 205.7) | **2.5 (1.7, 3.8)** | **<0.0001** |
| Cytology | 584 | 14 | 395.1 (234.0, 667.2) | 6816 | 67 | 162.9 (128.2, 207.0) | **2.4 (1.4, 4.3)** | **0.0026** |
| Baseline cytology negative | 566 | 10 | 290.3 (156.2, 539.5) | 6690 | 49 | 121.1 (92.5, 160.2) | **2.4 (1.2, 4.7)** | **0.0118** |
| Baseline cytology positive | 18 | 4 | 4078.0  (1530.5, 10865.8) | 126 | 18 | 2712.9 (1709.2, 4305.9) | 1.5 (0.51, 4.4) | 0.4609 |
| HPV | 624 | 16 | 487.1 (298.4, 795.2) | 6689 | 69 | 185.9 (146.9, 235.4) | **2.6 (1.5, 4.5)** | **0.0005** |
| Baseline HPV negative | 570 | 1 | 33.1 (4.7, 235.2) | 6339 | 16 | 45.8 (27.9, 74.4) | 0.73 (0.10, 5.5) | 0.7794 |
| Baseline HPV positive | 54 | 15 | 5625.1 (3391.2, 9330.7) | 350 | 53 | 2645.8 (2021.3, 3463.2) | **2.13 (1.2, 3.8)** | **0.0094** |
| CI, confidence interval; IR, incidence rate; IRR, incidence rate ratio; All population, women participating at baseline. | | | | | | | | |

| **Table R3.** Longitudinal test characteristics using cervical intraepithelial neoplasia grade 2 or worse (CIN2+) as outcome at 3, 5, 7 and 10 years of follow-up. | | | | | | | | |
| --- | --- | --- | --- | --- | --- | --- | --- | --- |
|  | Cumulative counts of CIN2+ at follow-up by baseline results | | | | Test Characteristics for CIN2+ | | | |
|  | Cytology | | HPV | | Cytology | | HPV | |
|  | Negative | Positive | Negative | Positive | Sensitivity (95% CI) | Specificity (95% CI) | Sensitivity (95% CI) | Specificity (95% CI) |
| Baseline | 7256 | 144 | 6909 | 404 |  |  |  |  |
| 3 years | 4 | 21 | 1 | 53 | 85.1  (76.6, 90.9) | 98.1  (98.1, 98.2) | 98.3  (88.7, 99.8) | 94.3  (94.2, 94.3) |
| 5 years | 21 | 21 | 3 | 65 | 52.1  (48.0, 56.3) | 97.8  (97.7, 97.8) | 96.0 *  (92.4, 97.9) | 92.1  (92.1, 92.2) |
| 7 years | 41 | 22 | 8 | 68 | 37.0  (34.1, 39.8) | 98.1  (98.0, 98.1) | 90.4 *  (87.4, 92.7) | 91.1  (91.0, 91.2) |
| 10 years | 58 | 22 | 16 | 68 | 29.2  (25.7, 33.0) | 97.9  (97.8, 98.1) | 82.4 *  (79.0, 85.3) | 92.2  (91.9, 92.4) |
| CI, confidence interval.  **P-*values for test of difference of proportions, compared to sensitivity of cytology at 5 years all *P*<0.0001 | | | | | | | | |

**Comment 19.**           Results: a.      It would be helpful if you could start with a description of how many were enrolled and any differences in important baseline characteristics across arms. The word median is used in almost every sentence in the beginning of the results. Please revise.

**Response:** Thanks for the comment. We have revised our results accordingly.

**Revised text in Results:**

**Page 14 lines 429-437** Among the 14,713 women included in the final analyses, there were 6909 with primary HPV negative result, 404 with primary HPV positive result, 7256 with primary cytology negative result, and 144 with primary cytology positive result (Fig 1). The average age of women at the time of recruitment was 58.1 years old. The median follow-up time for CIN2+ was 6.1 years in cytology arm and 5.0 years in HPV arm. For invasive cervical cancer, it was 9.8 years in both arms. The median number of screening tests was 2 for the whole cohort. The cytology arm had a median of 1 cytology test and 1 HPV test, while the HPV arm had 0 cytology tests and 2 HPV tests. Only 8.2% (1208/14713) of the whole cohort had a previous cervical abnormality (Table 1).

**Comment 20.**           Results: b.      I am worried about the use of sensitivity and specificity at 3, 5, 7, 10 years given that these estimates are all based on the baseline test result. In the clinical world, a clinician needs to know how sensitive and specific a test is to detect disease at the time of the test and not after 10 years. I think it is misleading. A lot can happen over the course of 10 years.

**Response:** Thanks for the comment. The longitudinal characteristics are indicators in the framework for evaluating screening strategies. It shows the ability of the primary testing method in detecting the presence of, or predict the development of cervical precancer, as certain HPV women do not have disease at the time of testing but can develop such in subsequent years [1]. The high sensitivity and specificity over time shows the additional test positivity in the HPV arm cannot be verified as just overdiagnosis, but seem to confirm the capacity of early detection of disease. The longitudinal characteritics can furthermore provide extra information about the primary test method than the sensitivity and specificity at the time of the test. We have revised our text in Method and Discussion sections to make the meaning of longitudinal characteristics more clear.

**Revised text in Method:**

**Page 11 lines 358-360** The longitudinal characteristics are important indicators in evaluating screening strategies and show the ability of the primary test method to detect or predict the presence or development of precancer [24].

**Revised text in Discussion:**

**Page 25 lines 642-644** We posit that the high sensitivity of primary HPV testing over a long time interval shows that high test positivity in the primary HPV arm was not merely overdiagnosis, but rather early detection of significant disease.

Reference

1. Adriaensen, Wim J., Cathy Matheï, Frank J. Buntinx, and Marc Arbyn. “A Framework Provided an Outline toward the Proper Evaluation of Potential Screening Strategies.” *Journal of Clinical Epidemiology* 66, no. 6 (June 2013): 639–47. <https://doi.org/10.1016/j.jclinepi.2012.09.018>.

**Comment 21.**           Results: c.      P 14, line 236-241: During follow-up?

**Response:** Thanks for the comment. We have revised the language.

**Revised text in Results:**

**Page 16 lines 453-456** We identified 17, 68, 59, and 22 cases of histopathology-confirmed CIN2+ among primary HPV negative, primary HPV positive, primary cytology negative, and primary cytology positive women during the follow-up, respectively. We identified 2 ICC cases in primary HPV negative women, 3 in primary HPV positive women, 13 in primary cytology negative women, and no ICC cases in primary cytology positive women during the follow-up.

**Comment 22.**           Discussion: a.      It would be nice if the first sentence had been stated earlier in the manuscript.

**Response:** Thanks for the comment. We have revised our abstract and results, and move this information forward in our manuscript.

**Revised text in Results:**

**Page 21 lines 508-511** Overall, women aged 56-61 in primary HPV arm had a tendency for a lower risk of invasive cervical cancer after 10 years of follow-up, compared to women in cytology arm in this trial (IRR: 0.39, 95% CI 0.14-1.09, *p*=0.0726, Table 4) and had a similar risk of CIN2+ (IRR: 1.1, 95% CI 0.8-1.5, *p*=0.5226, Table 4).

**Comment 23.**           Discussion: b.      I think it is important to discuss the risk of verification bias across groups. Only women who are referred for colposcopy and have biopsies collected are at risk.

**Response:** Thanks for the comment. The verification bias comes from the different follow-up strategies between groups of women. To make the whole screening working flow more clear, we added one process flowchart of screning in supporting information (S3 Figure). In our trial, the different follow-up of the two positive groups comes from the characteristics of HPV or cytology test. The HPV testing will lead to higher colposcopy referral rate. The difference is the key to prevent more invasive cancer in long term, and thus part of the causal mechanism of enhanced screening performance, rather than a bias. At baseline there were 78 women in HPV arm who should have been referred to colposcopy based on the screening protocal at that time and 76 of them had one histopathology result within one year after baseline test. In the cytology arm the number was 53 women who should have been referred to colposcopy and 49 had a histopathology result within one year. The compliance of the referal thus was high in both groups. According to the annual report of NKCx in 2019, the compliance of colposcopy referral after cytology positivity is 98% in Stockholm [1]. In addition, since 2017 all women in this trial started to receive exactly the same screening algorithm, namely primary HPV-based screening with a 7-year interval, which means there is no verification bias in negative groups after 2017. The only discrepancy we found is the time interval between the following screening test and baseline test. That difference only existed in sample taken in 2012 and 2013 and we have thus adjusted for sample year in our analysis. (Table 3 in S4 Text). The results remained very much stable.

Reference

1. Dillner, Joakim, Charlotte Örndal, and Pär Sparén. “Activity Report and Annual Report NKCx 2020.” Accessed August 27, 2024. <https://nkcx.se/templates/_rsrapport_2020.pdf>.

**Comment 24.**           Discussion: c.      P. 28, lines 412-414. Please revise to improve readability.

**Response:** Thanks for the comment. We have revised our language.

**Revised text in Discussion:**

**Page 28 lines 744-746** This suggests using primary HPV testing, risk stratification based on previous screening history and considering screen beyond 61 years old when releasing women from screening program.

**Comment 25.**           Discussion: d.      I think it would be interesting to discuss a bit more why you did not find any cancers in those with a positive cytology. Could it be because they were more likely to get treatment, thereby avoiding progression?

**Response:** Thanks for the comment. We did not find any cancer case after baseline screening and no cancer case appeared during the follow-up. This could suggest the clinical management after cytology positivity is adequate and provided women with proper treatment, thereby preventing progression. However, due to the limited number of positive cytology women (n=144), it is difficult to make any conclusive inference. We have revised our text and discussed it together with our findings in the HPV positive group.

**Revised text in Discussion:**

**Page 24 lines 597-601** Women with a cytology positive baseline result had 0 cases of ICC and women with HPV positive baseline result actually were diagnosed with 3 cases. This suggests the clinical management after cytology positivity was adequate and succeeded in preventing early lesions from progression. However, due to the limited number of cytology positive women (n=144), more evidence is needed.

**Comment 26.**            Figure 1. Please specify what "pathology" means. Cervical biopsy and LLETZ? Hysterectomy?

**Response:** Thanks for the comment. The ‘pathology’ means all histopathology test results, the sample could come from both biopsy or eg. LLETZ. We have revised the figure to avoid confusion (Fig 1).

**Comment 27.**            Figure 2. Please state which groups are being compared in the log-rank test. When looking at the figures, I don't see any differences in HPV neg vs cytology neg women in terms of CIN2+, but there is a major difference between those that are pos vs those that are negative. In terms of cancer, it is evident that HPV-pos have a high risk, while cytology positive have lowest. The supplementary figure illustrates what the title legend says: there is a significant difference in HPV neg vs cyt neg. Please revise.

**Response:** Thanks for the comment. The log-rank on the figure is the overall comparasion of all groups on each figure. We have revised and added another figure (Fig 3) to show the comparasion between negative groups and positive groups.

**Comment 28.**            Supplementary table 2. Confusing to read. You need to include the two groups you are comparing wo it becomes clear and concise to the reader which one is the reference group. Same goes for Supplementary table 3.

**Response:** Thanks for the comment. The reference group is now clearly stated in the table legend, see supporting information Table 2-3 in S4 Text.

**Reviewer #3:** Primary HPV testing had a higher sensitivity of detecting CIN2+ within a 7-year interval than primary cytology test within a 5-year interval (89.6% vs 50.9%).  
This study also showed that older women with an HPV negative result had higher risk of subsequent CIN2+ if they had a previous cytology positive test result. These results indicate that leaving with a cytology negative test at the age of 60 in general is not safe, given the high incidence rate of ICC  after 10 years, at least in women with previous abnormalities

**Response:** Many thanks for reviewing our manuscript and we appreciated the comments. According to your suggestions, we have carefully revised our manuscript. The detailed responses are listed below, point by point.

**Comment 1.**      Only 35% of countries worldwide 78 currently endorse HPV-based screening in their national recommendations. The recommended criterion of exiting program differs between women with only negative screening history within the past 10-year period, or women with one negative HPV test when arriving at the upper age limit. What is the case in most European countries? Can you please provide these data?

**Response:** Thanks for the comments. By the time of July 2019, there were only 5 European countries have already or started implementing primary HPV-based screening [1]. Based on the European guideline in 2015, in the HPV primary screening setting, the upper age limit is same as cytology primary screening (60 or 65 years), provided a women has a recent negative test [2]. We have revised our manuscript and added this information in the introduction section.

**Revised text in Introduction:**

**Page 6 lines 122-125** Regardless of the known low sensitivity of cytology-based cervical screening among older women [5], however, only 35% of countries worldwide currently endorse HPV-based screening in their national recommendations [6]. Only 5 European countries had started implementing primary HPV-based screening by 2019 [7].

**Page 6 lines 130-132** The European guidelines suggest women with one negative HPV test when arriving at the upper age limit (60 or 65 years) can discontinue screening [9].

Reference

1. Maver, P. J., and M. Poljak. “Primary HPV-Based Cervical Cancer Screening in Europe: Implementation Status, Challenges, and Future Plans.” *Clinical Microbiology and Infection* 26, no. 5 (May 1, 2020): 579–83. <https://doi.org/10.1016/j.cmi.2019.09.006>.
2. Karsa, Lawrence von, Marc Arbyn, Hugo De Vuyst, Joakim Dillner, Lena Dillner, Silvia Franceschi, Julietta Patnick, et al. “European Guidelines for Quality Assurance in Cervical Cancer Screening. Summary of the Supplements on HPV Screening and Vaccination.” *Papillomavirus Research* 1 (December 1, 2015): 22–31. <https://doi.org/10.1016/j.pvr.2015.06.006>.

**Comment 2.**      There is also a lack of evidence of when it is appropriate to exit the screening. Most countries recommend women exit screening around 60-70 years old. This study  supports the extending of age upper limit for cervical screening. Women with their last organized screening test above the  age of 55 still have a risk of invasive  cervical cancer higher than the elimination threshold. Till what age would the authors suggest screening? Would this be HPV-based, cytology based? Or a combination?

**Response:** Thanks for the comment. Based on current Swedish guidelines, the cervical screening is HPV-based and with an upper limit age of 64-70 years old based on when the last sample is taken. Our results support this guideline. We found that women who tested negative for cytology at the age of 56-61 still have high IR of ICC over 10 years of follow-up This suggests in the cytology-based screening, the upper limit age has to be increased to over 60 years old. In the HPV arm, we found one HPV negative screening test can lower the incidence rate (IR) of invasive cervical cancer (ICC) to 3.0/100,000 person-years (pys). In the subgroup analysis, women with one more screening around in HPV negative group had a IR of 1.9/100,000 pys, however, women without further screening after baseline still had a IR of 7.5/100,000 pys. This indicates multiple HPV negative tests could provide higher assurance against ICC in the long run. We currently do not have information to support the extension of upper limit age in a population that started HPV-based screening from a much younger age than our population, eg 30 years old. However, our data supports that in the era of transition from primary cytology to primary HPV, it would be safer to extend the upper limit age to over 60 years old. Our data favors HPV-based screening, however, we did not compare the effectiveness with HPV-based screening and co-testing, more evidence is needed to draw the conclusion. We have added relevant discussion in our manuscript.

**Revised text in Discussion:**

**Pages 25-26 lines 648-671** Our results support the current Swedish guideline for cervical screening, which recommends HPV-based screening with a 7-year interval for women aged over 50 years up until 64-70 years. We found that women who tested cytology negative at the age of 56-61 years still had a high IR of ICC over 10 years of follow-up, which indicates the upper limit age in any primary cytology-based screening needs to be increased to over 60 years. The increase of the upper limit age in HPV-based screening needs more consideration. Among women who tested baseline negative for HPV, we found they had an IR of ICC lower than the WHO’s elimination threshold, especially among women with another screening test after the trial baseline test. Women with one more organized screening test after baseline had a higher chance of being diagnosed with a precancer lesion and receive subsequent treatment. In contrast, among women without further screening after the trial baseline test, the IR of ICC remained high. In the era of transition from primary cytology testing to primary HPV testing, we think it would be safer to extend the upper limit age to over 60 years old. For women who start HPV-based screening at younger age (e.g. 30 years old), more evidence is needed to set up the upper limit screening age.

**Comment 3.**       This is one the first study that compared the longitudinal characteristics of HPV and cytology in detecting CIN2+ or ICC among older women by different time intervals. Table 3 shows longitudinal test characteristics using cervical intraepithelial neoplasia grade 2 or worse (CIN2+) as outcome at 3, 5, 7 and 10 years of followup. What would be the guideline interval? Based on what? Would this be different between women with previous abnormal smears?

**Response:** Thanks for the comment. Current Swedish guidelines suggest that the screening interval for women over 50 years old in HPV-based screening should be 7 years. Based on the low incidence rate of invasive cervical cancer over 10 years follow-up in HPV negative group, and the high longitudinal sensitivity of primary HPV testing, we think it would be safe to extend the screening interval to 7 years, potentially even 10 years for women over 50 years old. This in women with no previous abnormality.

However, some caution should be taken. In our cohort, only 570 women were baseline HPV-negative and had a previous abnormality, among whom we found 0 cancer cases over the 10-year follow-up. This amount of person-years may be too small to draw firm conclusions on intervals for this sub-group of women. Evidence from POBASCAM trial and our study both showed that women with abnormal screening history had a higher risk of CIN2+ during long-term follow-up [1]. More evidence is needed to ultimately determine the appropriate time interval for women with previous adverse screening history. We have revised our manuscript and added relevant information in the Discussion section.

**Revised text in Discussion:**

**Page 26 lines 673-679** The low IR of ICC over 10-year and the high longitudinal sensitivity of primary HPV testing support the extension of screening interval to 7 years, even to 10 years. However, evidence from both our study and the POBASCAM trial indicates that women with previous abnormal screening history have a higher risk of precancer lesion during long-term follow-up [32]. Risk stratification based on previous screening history may be motivated. Due to the limited number of ICC cases in our study, we were unable at this point to provide more stratified results on the risk of ICC among women with a previous abnormality.

Reference

1. Inturrisi, Federica, Lawrence Rozendaal, Nienke J. Veldhuijzen, Daniëlle A. M. Heideman, Chris J. L. M. Meijer, and Johannes Berkhof. “Risk of Cervical Precancer among HPV-Negative Women in the Netherlands and Its Association with Previous HPV and Cytology Results: A Follow-up Analysis of a Randomized Screening Study.” *PLoS Medicine* 19, no. 10 (October 2022): e1004115. <https://doi.org/10.1371/journal.pmed.1004115>.

**Comment 4.**      During the follow-up, women were  invited women with a negative result from HPV arm or cytology arm to the next test after 5 years, women with HPV positive and cytology-negative result were invited to a repeat HPV and cytology test after a  year (or after 3 years from May 2013); women with low-grade cytological abnormalities in  primary cytology test and negative HPV triage were invited for a repeat test one year later. Women with persistent HPV infection (positive for the same HPV type category), with lowgrade cytological abnormalities and HPV positive triage, or with CIN2+, were referred to colposcopy. Could you please pout these data in a process flowchart?

**Response:** Thanks for the comment. We have added one process flowchart for women who participated in this trial based on screening guidelines at that time in our supporting information (S3 Figure). We believe this will make the work flow of cervical screening more clear.

**S3 Figure.** **Process flowchart of cervical screening for women in this trial**. CIN2+, cervical intraepithelial neoplasia grade 2 or worse

**Reviewer #4:** This is a well conducted study on the effectiveness of primary HPV-based cervical screening  among older women from the long-term follow-up of a randomised healthcare policy trial on exit testing. The study design, statistical methods and analyses, and presentation and interpretation of the results are mostly adequate. However, there are still a few issues needing attention.

**Response:** We thank the reviewer for the supportive statement. According to your suggestions, we have carefully revised our manuscript. The detailed responses are listed below, point by point.

**Comment 1.** Throughout the paper, it hinted the HPV screening reduced the risk of cervical cancer, for example, in abstract, it says "Overall, women in the primary HPV arm had a tendency of a lower risk of ICC compared to women in cytology arm (IRR: 0.39; 95% CI: 0.14, 1.09)". However, the HPV screening is just diagnostic test which will not reduce the risk itself. It needs to be clear, it is the whole package - early and effective detection, early intervention, which may lead to reduction in the risk and improvement in the outcomes in the long term. It would be adquate to use 'associated' instead, and clearly discuss what exactly contribute the lower risk of ICC after the screening in the long run.

**Response:** Thanks for the comment. We have revised our introduction to make it more clear how screening can reduce the incidence of ICC. We also have revised our manuscript to avoid any direct using of decrease or reduce.

**Revised text in Introduction:**

**Page 6 lines 119-122** An efficient cervical cancer screening program can provide early detection of precancerous lesions which can lead to early treatment intervention and eventually reduction in risk of ICC; as well as providing early detection of manifest ICC.

**Revised text in Discussion:**

**Page 23 lines 555-557** Among women who tested negative for HPV, the long-term ICC incidence rate (3.0/100,000 person-years) was below the elimination threshold (4/100,000 person-years) [3].

**Page 23 lines 563-567** The overall long-term relative risk of ICC comparing primary HPV to primary cytology did not reach statistical significance in our current study, most likely due to a substantial part of the cytology arm subsequently being re-tested with the more sensitive method of primary HPV as a result of a change in national recommendations for screening among older women in 2017 – which affected all women equally in the nation.

**Comment 2.** Competing risk. As the outcome in the survival analyses is CIN2+ or ICC other than all cause mortality, there might be an issue of competing risk from death in the analyses. Need to know total number and percentage of death in the cohort during the follow up.

**Response:** Thanks for the comment. The NKCx database includes all tests related to cervical cancer, whether it is an organized screening program-derived test, opportunistic test, or symptom related indicated tests in the clinic. It also contains all the histopathology results related to the cervix. In the analysis of histopathology diagnosed CIN2+, the follow-up time is defined as the baseline to either the first histopathological diagnosis of CIN2+ or the date of the last registered test, including cytology, HPV, and histopathology before 31^st^ Dec 2022. We have revised our manuscript to make this clear. As long as there was a test registered, we assume that the woman was still alive and resident in Sweden. We only used death, emigration and hysterectomy as competing outcome when analyzing ICC as the outcome. We have added the number of deaths, hysterectomies and emigration in our flow chart (Fig 1).

**Revised text in Method:**

**Page 10 lines 316-321** We identified CIN2+ (includes CIN2, CIN3, AIS, ICC) diagnosed by a histopathology test in the NKCx. In the analysis of histopathology-confirmed CIN2+ as outcome, the follow-up time was defined as the time elapsed from the baseline test to either the first histopathological diagnosis of CIN2+, or the date of the last registered test, including cytology, HPV, and histopathology before 31^st^ Dec 2022.

**Comment 3.** Figure 2 is a key figure. For the 4 groups comparison results, the log rank test gives an overall significane of the comparison. However, we are also interested in those close curves, for example, in figure 2c, HPV postitive vs cytology positive, HPV negative vs cytology negative. Can authors please provide these mentioned test results too?

**Response:** Thanks for the comment. We have revised and added one figure (Fig 3) to show the comparasion between negative groups and positive groups and revised our results accordingly.

**Revised text in Results:**

**Page 16 lines 460-464** The cumulative incidence rates of CIN2+ and ICC are presented in Fig 2. No differences were found between two arms. The cumulative incidences of CIN2+ and ICC were all significantly higher among baseline primary cytology negative women compared to baseline primary HPV negative women (Fig 3, all *p<*0.05). No difference was detected among positive groups (Fig 3).

**Reviewer #5:** This is an important trial report on a topic for which there has been limited evidence to date - optimising cervical screening in older women. The setting for this longer-term observational follow-up of a trial is Sweden, where there is excellent capacity for case ascertainment and large-scale linkage capability. 

Women aged 56-61 were randomised in 2012-14, with samples randomised in the laboratory to cytology with HPV triage or HPV screening with cytological triage. HPV testing with HPV16/18 partial genotyping was used. Women with a negative result were invited to attend again in 5 years. A total of 14,719 women participated. 

Overall, the IRR for CIN2+ was similar for the two arms, but much lower in women with a negative baseline result of HPV. For ICC the IRR was significantly lower in HPV negative vs cytology negative women.
The longitudinal sensitivity of cytology clearly dropped over the follow-up time, whereas this was not the case to the same extent for HPV screening.

**Response:** Many thanks for reviewing our manuscript and the supportive statements on the setting of Sweden. According to your suggestions, we have carefully revised our manuscript. The detailed responses are listed below, point by point.

MAJOR COMMENTS
**Comment 1:**      It is surprising that a relatively small proportion of women at this age had a previous abnormality. Could more detail be provided about the look back period. Was lifetime screening behaviour or just very recent behaviour available (or something in between)? Were women who were previously unscreened considered differently from those who were well screened but without an abnormality? It is suggested the authors consider these issues for sensitivity analysis.

**Response:** Thanks for the comment. The definition of “previous abnormality” is based on availability of the registry data. The definition is thus receipt of any positive test results in NKCx before the baseline trial test, including cytology (ASCUS+), HPV positive, and histological confirmed CIN1+. The NKCx database includes all tests related to cervical cancer, whether it is an organized screening program-derived test, opportunistic test, or symptom related indicated tests in the clinic. It also contains all the histopathology results related to the cervix. The NKCx started to have complete coverage of the whole nation including the Stockholm region since 1995, which means most women would have a full screening history dating back around at least 17 years. As this proportion is thus not based on a lifetime screening behaviour, the proportion of the previous abnormality could be somewhat underestimated. However, Pankakoski and collegues reported that the cumulative incidence of 22 years among a cohort of women born between 1950-1965, cervical abnormalities that warranted referral (CIN1+) was about 5.4% in the Finnish population [1], which was close to the proportion of women had previous CIN1+ in our population (6.3%). We have revised out manuscript to make the definition about previous abnormality clear.

We acknowledge that we were unable to differentiate women without any screening tests/history from simply well-screened women without any previous abnormality. Sweden has had a well organized cervical screening program covering the whole population since 1970s and there is little chance that women over 50 years old would never have been screened in the country before 1995. Indeed, we only found 254 women without any previous screening history before baseline in our database. There is therefore little chance that such a limitation would materially affect the results but we anyway performed a sensitive analysis by excluding women without any previous history. The results are presented below (Table R4, Table R5). The results remained stable. We have thus added relevant information about women without any screening history in Table 1.

**Revised text in Method:**

**Page 10 lines 306-310** We used the Swedish National Cervical Screening Registry (NKCx), which has a full coverage nationally of cervical cancer screening results since 1995 [20], including all organized screening program tests, all non-organized/opportunistic tests, all indicated tests due to e.g. clinical symptoms, and all cervical histopathology tests and results, to retrieve all the screening tests and test results from the trial and subsequently during our follow-up.

**Revised text in Supporting information (S4 Text):**

**Supporting information S4 Page 1 lines 25-28** We categorized women with any previous positive test results, including HPV, cytology, and/or histopathological tests, since the availability of the register data (mostly after 1995) before the randomized trial as women with a previous abnormality.

Reference

1. Pankakoski, Maiju, Sirpa Heinävaara, Tytti Sarkeala, and Ahti Anttila. “High Lifetime Probability of Screen-Detected Cervical Abnormalities.” *Journal of Medical Screening* 24, no. 4 (December 2017): 201–7. <https://doi.org/10.1177/0969141316685740>.

| **Table R4.** Incidence rate, incidence rate ratio, and 95% CI of cervical intraepithelial neoplasia grade 2 or worse (CIN2+) and invasive cervical cancer (ICC) after excluding women without any previous screening history. | | | | | | | | | | |
| --- | --- | --- | --- | --- | --- | --- | --- | --- | --- | --- |
|  | CIN2+ | | | | | Invasive Cervical Cancer | | | | |
|  | n ^*^ | number of cases | IR (/100,000 person-years) | IRR | *P* value | n | number of cases | IR (/100,000 person-years) | IRR | *P* value |
| All population | 14165 | 153 | 186.0 (158.7, 217.9) |  |  | 14459 | 17 | 12.3 (7.64, 19.8) |  |  |
| Cytology | 7124 | 75 | 173.7 (138.5, 217.8) | Ref. |  | 7263 | 12 | 17.3 (9.8, 30.4) | Ref. |  |
| HPV | 7041 | 78 | 199.6 (159.9, 249.2) | 1.2 (0.84, 1.58) | 0.3894 | 7196 | 5 | 7.3 (3.0, 17.4) | 0.42 (0.15, 1.19) | 0.1031 |
| Women with negative baseline result | | | | |  |  |  |  |  |  |
| Cytology | 6992 | 56 | 131.8 (101.5, 171.3) | Ref. |  | 7126 | 12 | 17.6 (10.0, 31.0) | Ref. |  |
| HPV | 6662 | 16 | 43.4 (26.6, 70.8) | **0.33 (0.19, 0.57)** | <0.0001 | 6805 | 2 | 3.1 (0.77, 12.3) | **0.17 (0.04, 0.78)** | **0.0222** |
| Women with positive baseline result | | | | |  |  |  |  |  |  |
| Cytology | 132 | 19 | 2692.1  (1717.2, 4220.6) | Ref. |  | 137 | 0 | 0 | Ref. |  |
| HPV | 379 | 62 | 2835.6  (2210.8, 3637.1) | 1.1 (0.63, 1.8) | 0.8430 | 391 | 3 | 81.3 (26.2, 251.9) | ∞ | - |
| *n for CIN2+ exclude women with histopathological diagnosed CIN2+ before the randomized trial; CI, confidence interval; IR, incidence rate; IRR, incidence rate ratio; ref, reference; all population, women participating at baseline; | | | | | | | | | | |

| **Table R5.** IR, IRR and 95% CI of cervical intraepithelial neoplasia grade 2 or worse (CIN2+) and invasive cervical cancer (ICC) of women with previous abnormality compared to women without previous abnormality after excluding women without any previous screening history | | | | | | | | |
| --- | --- | --- | --- | --- | --- | --- | --- | --- |
|  | **With a previous abnormality** | | | **Without a previous abnormality** | | | **IRR** | ***P* Value** |
|  | n ^*^ | number of cases | IR (/100,000 person-years) | n ^*^ | number of cases | IR (/100,000 person-years) |  |  |
| **CIN2+** |  |  |  |  |  |  |  |  |
| All population | 914 | 22 | 430.2 (283.3, 653.3) | 13251 | 131 | 169.8 (143.1, 201.5) | **2.5 (1.6, 4.0)** | **<0.0001** |
| Cytology | 445 | 12 | 449.6 (255.3, 791.7) | 6679 | 63 | 155.5 (121.5, 199.0) | **2.9 (1.6, 5.4)** | **0.0007** |
| Baseline cytology negative | 432 | 9 | 345.5 (179.8, 664.1) | 6560 | 47 | 345.5 (179.8, 664.1) | **2.9 (1.4, 6.0)** | **0.0031** |
| Baseline cytology positive | 13 | 3 | 4681.9 (1510.0, 14516.8) | 119 | 16 | 2493.4 (1527.5, 4070.0) | 1.9 (0.6, 6.4) | 0.3166 |
| HPV | 469 | 10 | 408.9 (220.0, 760.0) | 6572 | 68 | 185.6 (146.4, 235.4) | **2.2 (1.1, 4.3)** | **0.0197** |
| Baseline HPV negative | 427 | 0 | 0 | 6235 | 16 | 46.2 (28.3, 75.4) | 0 | 0.9998 |
| Baseline HPV positive | 42 | 10 | 4733.2 (2546.7, 8797.1) | 337 | 52 | 2632.7 (2006.1, 3454.9) | 1.8 (0.9, 3.5) | 0.0893 |
| **Invasive Cervical Cancer** |  |  |  |  |  |  |  |  |
| All population | 1208 | 2 | 17.4 (4.4, 69.7) | 13251 | 15 | 11.8 (7.1, 19.6) | 1.5 (0.34, 6.44) | 0.6074 |
| Cytology | 584 | 0 | 0 | 6679 | 12 | 18.8 (10.7, 33.1) | 0 | 0.9997 |
| Baseline cytology negative | 566 | 0 | 0 | 6560 | 12 | 19.1 (10.9, 33.7) | 0 | 0.9997 |
| Baseline cytology positive | 18 | 0 | 0 | 119 | 0 | 0 | ∞ | 1.0000 |
| HPV | 624 | 2 | 33.8 (8.4, 135.1) | 6572 | 3 | 4.8 (1.5, 14.8) | **7.1 (1.2, 42.4)** | **0.0319** |
| Baseline HPV negative | 570 | 0 | 0 | 6235 | 2 | 3.4 (0.84, 13.4) | 0 | 0.9999 |
| Baseline HPV positive | 54 | 2 | 407.7 (102.0, 1630.1) | 337 | 1 | 31.2 (4.4, 221.8) | **13.1 (1.2, 143.9)** | **0.0359** |
| *calculation related to CIN2+ exclude women with histopathological diagnosed CIN2+ before the randomized trial; CI, confidence interval; IR, incidence rate; IRR, incidence rate ratio; All population, women participating at baseline. | | | | | | | | |

**Comment 2:**      The moderate imbalance of follow-up between the two study arms is potentially an issue because in this observational follow-up design there is no consistent ascertainment of whether disease is present at the end of follow-up. Therefore, ascertainment bias for the outcome of invasive cervical cancer (as well as CIN2+ and CIN3+) is a possibility especially in respect to early stage non-symptomatic cancers. It is somewhat reassuring that biopsy rates appeared fairly similar. However, the IRs of ICC tended towards being different in women who received an organised test after the baseline test than those that did not. Could this latter issue be discussed and explained in more detail?

**Response:** Thanks for the comment. We acknowledge that there was no per-protocol ascertainment test at the end of this trial, however, the subsequent follow-up time was generally long which gave ample opportunity for potential short-term imbalances in ascertainment to even out.

This since the median follow-up time for invasive cervical cancer was 9.8 years, yet the imbalance of the two studies occurred already in the 3-5 years after the baseline test. Ergo, after said imbalance we still have nearly 5 years of follow-up for women, which we posit gave enough time for us to detect invasive cancer cases. In the cytology arm, the women received more (HPV-based) screening than originally planned with shorter time interval, which enabled earlier detection of precancer lesion. In Fig 3, we can clearly see the increase of CIN2+ incidence in cytology negative group occurs earlier than in the HPV negative group. This would actually result in an underestimation of the effectiveness of primary HPV testing when compared to primary cytology testing. We previously alluded to this in Discussion (page 22 lines 677-688).

To alleviate this concern, we performed a sensitive analysis among women with organized screening test and without organized screening test after baseline. Because it is a subgroup analysis and the number of cancer cases was sparse, the interpretation should be with some caution. One explanation could be women with one more organized screening test after baseline had higher chance to find precancer and get treatment. We have revised our discussion accordingly.

**Revised text in Discussion:**

**Pages 25-26 lines 653-667** Among women who tested baseline negative for HPV, we found they had an IR of ICC lower than the WHO’s elimination threshold, especially among women with another screening test after the trial baseline test. Women with one more organized screening test after baseline had a higher chance of being diagnosed with a precancer lesion and receive subsequent treatment. In contrast, among women without further screening after the trial baseline test, the IR of ICC remained high.

**Comment 3:**      Can explicit detail be provided on the 18 cancers on the mode of detection - especially Stage/sub-stage at diagnosis and whether symptomatic or screen-detected. A table could ideally be provided on this showing classifications for each cancer. It is important to know which cancers are screen 'successes' i.e. screen/triage/fu detected, or screen 'failures' i.e. interval cancers detected symptomatically.

**Response:** Thanks for the comment. We appreciate that this is an important reviewer issue and have in this Response letter below provided a table (Table R6) concerning the 18 cancers, inculding the stage at diagnosis and whether they are screen detected or not. As can be seen, the small sample numbers mean few firm conclusions can be drawn at this point. Please note: this is not public data and cannot be shared in the public realm due to GDPR concerns. We only share it here to be transparent with our data in response to the reviewer’s justified question.

| **Table R6**.Number of invasive cervical cancer (ICC) cases based on baseline test result, detection mode, and stage at diagnosis. | | | | |
| --- | --- | --- | --- | --- |
| Detection mode | Stage at diagnosis | Cytology negative | HPV negative | HPV positive |
| Screen detected | IA | 2 | 0 | 1 |
|  | IB | 3 | 1 | 0 |
|  | II+ | 1 | 0 | 0 |
| Symptomatic detected | IA | 0 | 0 | 0 |
|  | IB | 2 | 0 | 0 |
|  | II+ | 5 | 1 | 2 |
| *Screen detected: detected by organized screening test, includig primary test, triage test, and follow-up test; Symptomatic detected: detected by opportunistic/sympton-indicative test. | | | | |

**Comment 4:**      No detail on cancer stage is provided but microinvasive cancers would normally be only screen detected and it would be good to see all the results also presented with these excluded and with a frank cancer outcome only. Is staging at diagnosis data available? If not, this reason for this data limitation should be explained and the implications discussed as a limitation of the analysis.

**Response:** Please see response above. Due to the small number of cancer cases, we do not think it is appropriate to do any analysis based on a frank cancer outcome. The results are too imprecise and could be misleading. And due to GDPR concerns, we cannot publish this table in our manuscript. Since we cannot publish this data we have added a limitation as suggested.

**Revised text in Discussion:**

**Page 27 lines 728-731** Finally, because of the small number of ICC cases in our study, for reasons of data privacy we could not present details on stage and whether the cancer was screening- or symptom detected. This should remain the focus of future work.

**Comment 5:**      Particularly, given that the IR of ICC in women with a positive HPV test was apparently quite high (albeit with only three cases and thus high uncertainty in the estimate) it is important to provide more detail on the three cancers in this group? If there was evidence that these cases represented early stage microinvasive cancers rather than frank cancers and they were screen-detected, then the inference could be made that these were detected earlier because of screening.

**Response:** Please see responses above to comment 3 and 4.

**Comment 6:**      The elimination threshold is not really designed to be used in the way it is here - i.e. as a threshold for the implied safety of discharge of women. Elimination is a population-wide average rate and population-wide target. Although it is an interesting to compare outcomes in groups here to elimination rates, I would suggest not relying on this for the primary conclusions. The acceptability of screening discharge in a population subgroup post-testing does depend on the rate of disease thereafter but the post-test probability should be compared to the disease rates for women routinely referred to colposcopy, follow-up, or routine screening in that setting. Different countries have different acceptable disease rates for these thresholds.

**Response:** Thank you for the careful consideration. We agree that the elimination threshold is a population-wide average rate, and countries may decide their own threshold for varied groups. We used the incidence rates to discuss/conceptualize whether a group can safely exit screening, which we respectfully think is not inappropriate. Indeed, we posit that it is highly useful in the current context, because: a. the test negative group, especially aiming to discountinue screening, should have an incidence rate below the elimination average, which “allows” other groups to have higher; and b. as a country with a well organized cervical screening and prevention program, one is obligaged to aim for the highest standard of elimination; and c. to provide evidence to guidelines, a standard on absolute risk is informative in addition to relative risk.

MINOR COMMENTS
**Comment 7:**      The second peak referred to in the first sentence of the introduction is most likely because of screening stopping and hence varies by screening stopping age in a population - this should be made clearer.

**Response:** Thanks for the comment. The primary test method, time interval, and age to stop screening are all important aspects of screening program.We have revised the introduction and be more specific about the suboptimal screening program design.

**Revised text in Introduction:**

**Page 6 lines 116-119** The relatively higher incidence of cervical cancer and cervical intraepithelial neoplasia grade 2 or worse (CIN2+) among older women has been associated with suboptimal screening participation, diagnostic difficulties due to morphological changes due to mucosal atrophy due to menopause, and controversy in screening upper age limit [1,4].

**Comment 8:**      In Table 1, are the non-screening tests including both triage and follow-up tests? Can this be made clearer or broken down?

**Response:** Thanks for the comment. We identify different test by the screen type (screening test and non-screening test) and sample date in NKCx. The non-screening tests only include opportunistic test or indicative test taken because of clinical symptons. The follow-up tests for women tested positive were included in the screening test. The triage tests haven’t been calculated individually because the traige tests share the same sample date as the primary test. We reused the original sample to do the triage test. We have made it clear in the footnote of Table 1.

General editorial requests:
(Note: not all will apply to your paper, but please check each item carefully)

*       We ask every co-author listed on the manuscript to fill in a contributing author statement, making sure to declare all competing interests. If any of the co-authors have not filled in the statement, we will remind them to do so when the paper is revised. If all statements are not completed in a timely fashion this could hold up the re-review process. If new competing interests are declared later in the revision process, this may also hold up the submission. Should there be a problem getting one of your co-authors to fill in a statement we will be in contact. Please do not add or remove authors without first discussing this with the handling editor. You can see our competing interests policy here: [https://eur01.safelinks.protection.outlook.com/?url=http%3A%2F%2Fjournals.plos.org%2Fplosmedicine%2Fs%2Fcompeting-interests&data=05%7C02%7Cqingyun.yao%40ki.se%7C276d3bbccfae481bcb3a08dcafb91455%7Cbff7eef1cf4b4f32be3da1dda043c05d%7C0%7C0%7C638578456932458124%7CUnknown%7CTWFpbGZsb3d8eyJWIjoiMC4wLjAwMDAiLCJQIjoiV2luMzIiLCJBTiI6Ik1haWwiLCJXVCI6Mn0%3D%7C0%7C%7C%7C&sdata=qV9i0rbWfi8NOZ5y1MZ%2FvhfH2wEG4OkHX5WzlJBkMH4%3D&reserved=0](http://journals.plos.org/plosmedicine/s/competing-interests).

**Response:** We will remind all the co-authors to declare competing interests. Thanks for the reminder.

*       Please upload any figures associated with your paper as individual TIF or EPS files with 300dpi resolution at resubmission; please read our figure guidelines for more information on our requirements: [https://eur01.safelinks.protection.outlook.com/?url=http%3A%2F%2Fjournals.plos.org%2Fplosmedicine%2Fs%2Ffigures&data=05%7C02%7Cqingyun.yao%40ki.se%7C276d3bbccfae481bcb3a08dcafb91455%7Cbff7eef1cf4b4f32be3da1dda043c05d%7C0%7C0%7C638578456932462220%7CUnknown%7CTWFpbGZsb3d8eyJWIjoiMC4wLjAwMDAiLCJQIjoiV2luMzIiLCJBTiI6Ik1haWwiLCJXVCI6Mn0%3D%7C0%7C%7C%7C&sdata=E8ZA2WMC%2FclooJzu6cTXmOXsic7ofTejcSeCRLQicak%3D&reserved=0](http://journals.plos.org/plosmedicine/s/figures). While revising your submission, please upload your figure files to the PACE digital diagnostic tool, [https://eur01.safelinks.protection.outlook.com/?url=https%3A%2F%2Fpacev2.apexcovantage.com%2F&data=05%7C02%7Cqingyun.yao%40ki.se%7C276d3bbccfae481bcb3a08dcafb91455%7Cbff7eef1cf4b4f32be3da1dda043c05d%7C0%7C0%7C638578456932466083%7CUnknown%7CTWFpbGZsb3d8eyJWIjoiMC4wLjAwMDAiLCJQIjoiV2luMzIiLCJBTiI6Ik1haWwiLCJXVCI6Mn0%3D%7C0%7C%7C%7C&sdata=4vaMvbWx8aMvCuwdvvvK%2F57MmcXE9%2B75d1tcC%2BQSDxg%3D&reserved=0](https://pacev2.apexcovantage.com/). PACE helps ensure that figures meet PLOS requirements. To use PACE, you must first register as a user. Then, login and navigate to the UPLOAD tab, where you will find detailed instructions on how to use the tool. If you encounter any issues or have any questions when using PACE, please email us at [PLOSMedicine@plos.org](mailto:PLOSMedicine@plos.org).

**Response:** We have revised the figures and uploaded them separately based on the requirements of PLOS medicine.

*       Please ensure that the paper adheres to the PLOS Data Availability Policy (see [https://eur01.safelinks.protection.outlook.com/?url=http%3A%2F%2Fjournals.plos.org%2Fplosmedicine%2Fs%2Fdata-availability&data=05%7C02%7Cqingyun.yao%40ki.se%7C276d3bbccfae481bcb3a08dcafb91455%7Cbff7eef1cf4b4f32be3da1dda043c05d%7C0%7C0%7C638578456932469951%7CUnknown%7CTWFpbGZsb3d8eyJWIjoiMC4wLjAwMDAiLCJQIjoiV2luMzIiLCJBTiI6Ik1haWwiLCJXVCI6Mn0%3D%7C0%7C%7C%7C&sdata=KkUSQrCGn%2FDN%2F4E1ynd96PZXuU3Vu%2B3pDTkDehn33es%3D&reserved=0)](http://journals.plos.org/plosmedicine/s/data-availability), which requires that all data underlying the study's findings be provided in a repository or as Supporting Information. For data residing with a third party, authors are required to provide instructions with contact information (web or email address) for obtaining the data. Please note that a study author cannot be the contact person for the data. PLOS journals do not allow statements supported by "data not shown" or "unpublished results." For such statements, authors must provide supporting data or cite public sources that include it.

**Response:** Our data contains potentially identifying patient information. We have added the Data Availability Statement in our manuscript. The data is deposited with the Swedish Cervical Screening Registry ([www.nkcx.se](http://www.nkcx.se)) and can be requested at [info@nkcx.se](mailto:info@nkcx.se) (administrator Sara Nordqvist Kleppe). One can vist <http://www.nkcx.se/research_e.htm> for detailed information about how to apply for access.

*       We expect all researchers with submissions to PLOS in which author-generated code underpins the findings in the manuscript to make all author-generated code available without restrictions upon publication of the work. In cases where code is central to the manuscript, we may require the code to be made available as a condition of publication. Authors are responsible for ensuring that the code is reusable and well documented. Please make any custom code available, either as part of your data deposition or as a supplementary file. Please add a sentence to your data availability statement regarding any code used in the study, e.g. "The code used in the analysis is available from Github [URL] and archived in Zenodo [DOI link]" Please review our guidelines at <https://eur01.safelinks.protection.outlook.com/?url=https%3A%2F%2Fjournals.plos.org%2Fplosmedicine%2Fs%2Fmaterials-software-and-code-sharing&data=05%7C02%7Cqingyun.yao%40ki.se%7C276d3bbccfae481bcb3a08dcafb91455%7Cbff7eef1cf4b4f32be3da1dda043c05d%7C0%7C0%7C638578456932475283%7CUnknown%7CTWFpbGZsb3d8eyJWIjoiMC4wLjAwMDAiLCJQIjoiV2luMzIiLCJBTiI6Ik1haWwiLCJXVCI6Mn0%3D%7C0%7C%7C%7C&sdata=RKlafUgobUxl6KJxQQxDuugMo6JcNw5MEvSYuuVu4OQ%3D&reserved=0> and ensure that your code is shared in a way that follows best practice and facilitates reproducibility and reuse. Because Github depositions can
be readily changed or deleted, we encourage you to make a permanent DOI'd copy (e.g. in Zenodo) and provide the URL.

**Response:** Our statistical analysis is a standard follow-up of a randomized trial. The code is not the central of the manuscript. The code can be shared at request.

Please review our guidelines

*       DATA AVAILABILITY: The Data Availability Statement (DAS) requires revision. Please clarify whether the data are freely/publicly available, owned by a third party but freely available upon request, or not freely available (if so, please briefly describe the ethical, legal, or contractual restriction that prevents you from sharing the data).

**Response:** Our data contains potentially identifying patient information, thus according to Swedish law the authors are not able to make the dataset publicly available. The data is deposited with the Swedish Cervical Screening Registry (www.nkcx.se) and can be requested at info@nkcx.se (administrator Sara Nordqvist Kleppe). One can vist http://www.nkcx.se/research_e.htm for detailed information about how to apply for access. We have given the above-mentioned information in the data availability.

*       COMPETING INTEREST: All authors must declare their relevant competing interests per the PLOS policy, which can be seen here: [https://eur01.safelinks.protection.outlook.com/?url=https%3A%2F%2Fjournals.plos.org%2Fplosmedicine%2Fs%2Fcompeting-interests&data=05%7C02%7Cqingyun.yao%40ki.se%7C276d3bbccfae481bcb3a08dcafb91455%7Cbff7eef1cf4b4f32be3da1dda043c05d%7C0%7C0%7C638578456932480152%7CUnknown%7CTWFpbGZsb3d8eyJWIjoiMC4wLjAwMDAiLCJQIjoiV2luMzIiLCJBTiI6Ik1haWwiLCJXVCI6Mn0%3D%7C0%7C%7C%7C&sdata=h83qUkoNO7ojq4vzZp7cCBYt5gE8XoLNyNsjAbJy5to%3D&reserved=0](https://journals.plos.org/plosmedicine/s/competing-interests)
For authors with ties to industry, please indicate whether any of the interests has a financial stake in the results of the current study.

**Response:** We will remind all the co-authors to declare competing interests.

*       Please include the completed CONSORT checklist as Supporting Information. When completing the checklist, please use section and paragraph numbers, rather than page numbers.

**Response:** We have included CONSORT checklist as Supporting information and completed the checklist based on section and paragraph numbers.

FORMATTING - GENERAL
*       Abstract: Please structure your abstract using the PLOS Medicine headings (Background, Methods and Findings, Conclusions). Please combine the Methods and Findings sections into one section.

**Response:** We have reformated our abstract accordingly.

*       At this stage, we ask that you include a short, non-technical Author Summary of your research to make findings accessible to a wide audience that includes both scientists and non-scientists. The Author Summary should immediately follow the Abstract in your revised manuscript. This text is subject to editorial change and should be distinct from the scientific abstract. Ideally each sub-heading should contain 2-3 single sentence, concise bullet points containing the most salient points from your study. In the final bullet point of 'What Do These Findings Mean?', please include the main limitations of the study in non-technical language. Please see our author guidelines for more information: [https://eur01.safelinks.protection.outlook.com/?url=https%3A%2F%2Fjournals.plos.org%2Fplosmedicine%2Fs%2Frevising-your-manuscript%23loc-author-summary&data=05%7C02%7Cqingyun.yao%40ki.se%7C276d3bbccfae481bcb3a08dcafb91455%7Cbff7eef1cf4b4f32be3da1dda043c05d%7C0%7C0%7C638578456932484946%7CUnknown%7CTWFpbGZsb3d8eyJWIjoiMC4wLjAwMDAiLCJQIjoiV2luMzIiLCJBTiI6Ik1haWwiLCJXVCI6Mn0%3D%7C0%7C%7C%7C&sdata=GwHAfFVzmoPUrBRMZsbpQEu4m4VIqAWmeGx6myKx%2F64%3D&reserved=0](https://journals.plos.org/plosmedicine/s/revising-your-manuscript#loc-author-summary).

**Response:** We have included a short, non-technical author summary after the abstract.

*       Please express the main results with 95% CIs as well as p values. When reporting p values please report as p<0.001 and where higher as the exact p value p=0.002, for example. Throughout, suggest reporting statistical information as follows to improve clarity for the reader "22% (95% CI [13%,28%]; p</=)". Please be sure to define all numerical values at first use.

**Response:** We have reported the main results with 95% Cis as well as p values, and we have revised our text and table accordingly.

*       Please include page numbers and line numbers in the manuscript file. Use continuous line numbers (do not restart the numbering on each page).

**Response:** We have reformated our abstract accordingly.

*       Please cite the reference numbers in square brackets. Citations should precede punctuation.

**Response:** We have reformated our abstract accordingly.

FIGURES AND TABLES
*       Please provide titles and legends for all figures and tables (including those in Supporting Information files).

**Response:** We have added titles and legends for all figures and tables.

*       Please define all abbreviations used in each figure/table (including those in Supporting Information files).

**Response:** We have defined all abbreviations in all figures and tables.

*       Please consider avoiding the use of red and green in order to make your figure more accessible to those with color blindness.

**Response:** We have revised our figures and made them into black and white.

SUPPLEMENTARY MATERIAL
*       Please note that supplementary material will be posted as supplied by the authors. Therefore, please amend it according to the relevant comments outlined here.

**Response:** We have amended our supporting information accordingly.

*       Please cite your Supporting Information as outlined here: [https://eur01.safelinks.protection.outlook.com/?url=https%3A%2F%2Fjournals.plos.org%2Fplosmedicine%2Fs%2Fsupporting-information&data=05%7C02%7Cqingyun.yao%40ki.se%7C276d3bbccfae481bcb3a08dcafb91455%7Cbff7eef1cf4b4f32be3da1dda043c05d%7C0%7C0%7C638578456932488974%7CUnknown%7CTWFpbGZsb3d8eyJWIjoiMC4wLjAwMDAiLCJQIjoiV2luMzIiLCJBTiI6Ik1haWwiLCJXVCI6Mn0%3D%7C0%7C%7C%7C&sdata=PBhup7RkrK7HArGnVo9n6cYi%2F1dlMn18jb5BlFMV2BM%3D&reserved=0](https://journals.plos.org/plosmedicine/s/supporting-information)

**Response:** We have cited our supporting information accordingly.

REFERENCES
*       PLOS uses the numbered citation (citation-sequence) method and first six authors, et al.

**Response:** Thanks for the comment. We have reformated our references accordingly.

*       Please ensure that journal name abbreviations match those found in the National Center for Biotechnology Information (NCBI) databases ([https://eur01.safelinks.protection.outlook.com/?url=http%3A%2F%2Fwww.ncbi.nlm.nih.gov%2Fnlmcatalog%2Fjournals&data=05%7C02%7Cqingyun.yao%40ki.se%7C276d3bbccfae481bcb3a08dcafb91455%7Cbff7eef1cf4b4f32be3da1dda043c05d%7C0%7C0%7C638578456932493505%7CUnknown%7CTWFpbGZsb3d8eyJWIjoiMC4wLjAwMDAiLCJQIjoiV2luMzIiLCJBTiI6Ik1haWwiLCJXVCI6Mn0%3D%7C0%7C%7C%7C&sdata=%2BGZRrdhs3ytkvMlBjLKdDdBpRC1kt6NEkIz%2BtaxIfmc%3D&reserved=0](http://www.ncbi.nlm.nih.gov/nlmcatalog/journals)), and are appropriately formatted and capitalised.

**Response:** We have checked our references and make sure the abbreviations are right.

*       Where website addresses are cited, please include the complete URL and specify the date of access (e.g. [accessed: 12/06/2023]).

**Response:** We have revised our references accordingly

*       Please also see [https://eur01.safelinks.protection.outlook.com/?url=https%3A%2F%2Fjournals.plos.org%2Fplosmedicine%2Fs%2Fsubmission-guidelines%23loc-references&data=05%7C02%7Cqingyun.yao%40ki.se%7C276d3bbccfae481bcb3a08dcafb91455%7Cbff7eef1cf4b4f32be3da1dda043c05d%7C0%7C0%7C638578456932497949%7CUnknown%7CTWFpbGZsb3d8eyJWIjoiMC4wLjAwMDAiLCJQIjoiV2luMzIiLCJBTiI6Ik1haWwiLCJXVCI6Mn0%3D%7C0%7C%7C%7C&sdata=Gcs0LOtSlZ49Lw2Ybm09Pl3r%2BzZGE](https://journals.plos.org/plosmedicine/s/submission-guidelines#loc-references)VSNSYD9R%2Fv3iFU%3D&reserved=0 for further details on reference formatting.

**Response:** We have checked the reference formatting and tried our best to ensure the correct formatting in reference.

STUDY TYPE-SPECIFIC REQUESTS
*       Please report your study according to CONSORT, but explicitly state that it is a sub-study. Please ensure that the Abstract describes the main points of the original trial (2-3 sentences, including study population, study dates, intervention and primary outcome), but that the main part of the Abstract describes the details of the follow-up study.

**Response:** Please note that this is not a sub-study. The original design of the trial had two main phases, which we have now clarified in the abstract and introduction. We have reported our study accoring to CONSORT. We have added relevant information in the abstract accordingly.

*       Please ensure that study registration details are included in the Methods section.

**Response:** We have added registration details in the Methods section.

*       Abstract: Please include the study design, population and setting, number of participants, years during which the study took place (original enrollment and follow up), length of follow up, and main outcome measures.

**Response:** We have included above-mentioned information in our abstract.

*       Please include absolute numbers wherever you report percentages; eg, n/N (%)

**Response:** We have checked and revised our manuscript and included absolute number when report percentages.

*       In keeping with our commitment to Open Science, please include the study protocol document and analysis plan (including any amendments) as Supporting Information to be published with the manuscript if accepted.

**Response:** We have included the study protocol and analysis plan as supporting information.
