# Supplementary material for: Evaluation of primary HPV-based cervical screening among older women: Long-term follow-up of a randomized healthcare policy trial in Sweden
Source: PLoS Med. 2024 Dec 19;21(12):e1004505. doi: 10.1371/journal.pmed.1004505 (PMC11706452; doi:10.1371/journal.pmed.1004505)
Supplement: Attachment — Submitted filename: Response to Reviewers_R2_final.docx [file pmed.1004505.s008.docx]

**Response to Reviewers: Effectiveness of primary HPV-based cervical screening among older women: Long-term follow-up of a randomised healthcare policy trial**

Dear Editors,

We are grateful for the reviewers’ positive feedback on our previous revision. And thank you for considering our manuscript for publication. We are pleased to note that they found the manuscript improved and have no further comments. In this revision, we have carefully revised our paper according to the editors' requests. The detailed responses are liste below, point by point.

***Please note that all the line numbers and page numbers cited below are as appear in the fully expanded track change version of the revised manuscript.***

Comments from Academic Editor:

The authors seem to have responded well to a number of questions and issues raised by the reviewers. The large size, initial randomisation and long follow-up make this paper to some extent unique, and the findings are important and have direct clinical implications. The statistically significant effects of screening modality on CIN2+ and ICC are meaningful, but so is the trend in the overall population, which did not reach statistical significance but has a confidence interval that shows strong evidence of an overall benefit.

**Response:** We thank Academic Editor for the supportive feedback.

Requests from Editors:

TITLE
We suggest changing the title to “Evaluation of primary HPV-based cervical screening among older women: Long-term follow-up of a randomised healthcare policy trial in Sweden”

**Response:** Thank you. We have revised the title accordingly.

**Revised text in Title page:**

**Page 1 lines 1-2** Evaluation of primary HPV-based cervical screening among older women: Long-term follow-up of a randomised healthcare policy trial in Sweden

CODE AVAILABILITY
Thank you for stating that code can be shared at request. Could you provide an appropriate contact (web or email address) for inquiries?

**Response:** Thank you. We have included the email address ([Qingyun.yao@ki.se](mailto:Qingyun.yao@ki.se)) in the Data availability statement as the contact for code-sharing inquiries.
**Revised text in Data availability:**

**Page 30 lines 672-677** Our data contains potentially identifying patient information, thus according to Swedish law the authors are not able to make the dataset publicly available. The data is deposited with the Swedish Cervical Screening Registry (www.nkcx.se) and can be requested at info@nkcx.se (administrator Sara Nordqvist Kleppe). One can vist http://www.nkcx.se/research_e.htm for detailed information about how to apply for access. The code used in the analysis is available at request from Qingyun Yao (qingyun.yao@ki.se).

ABSTRACT
1) l.39: Please define ‘HPV’ at first use.

**Response:** We have revised accordingly.

**Revised text in Abstract:**

**Page 3 lines 41-42** Evidence on invasive cervical cancer prevention among older women is limited, especially with the introduction of Human Papillomavirus (HPV)-based screening and longer interval.

2) l. 51: Do you mean ‘primary HPV testing’?

**Response:** Thank you for the comment. Participants were randomized into the primary HPV arm and the primary cytology arm based on the last digit of their personal identity number. 1) Cytology arm: primary cytological test and with triage HPV analysis for women with low-grade cytological abnormality (atypical squamous cells of undetermined significance, ASCUS, or CIN1/low-grade squamous intraepithelial lesion, LSIL), according to the routine program; and 2) HPV-arm: primary HPV test and triage cytological analysis for women with positive HPV results. To avoid confusion, we have revised the terminology throughout the manuscript. Instead of referring to "primary HPV" or "primary cytology" alone, we now consistently use "primary HPV-based screening"/"primary cytology-based screening" and “primary HPV arm”/ “primary cytology arm”or specify "primary HPV test" and "primary cytology test" when referring to the respective procedures.

**Revised text in Abstract:**

**Page 3 lines 52-55** The initial phase of the trial, which was the focus of this study, targeted women aged 56-61 years old in 2012-2014 who were randomized to primary cytology arm (n=7,401) or primary HPV arm (n=7,318).

3) l.57: Please refrain from describing non-significant results as showing “a tendency for”. Please revise throughout the manuscript.

**Response:** Thank you for the comment. We have revised throughout our manuscript for the expression to avoid using ‘a tendency for’.

**Revised text in Abstract:**

**Page 3 lines 63-67** We further found that the overall point estimate for the risk of ICC over 10 years of follow-up among women in the primary HPV arm was 0.39 compared to women in the primary cytology arm, but this was not statistically significant (IRR: 0.39; 95% confidence interval, CI [0.14, 1.09]; *p*=0.0726).

**Revised text in Discussion:**

**Page 24 lines 493-496** We performed a long-term evaluation of women ages 56-61 who received a primary HPV-based screening in 2012-2014 and followed their risk of invasive cervical cancer over 10 years of follow-up, compared to women who received primary cytology-based screening within the same trial.

4) l.58: Please define ‘CI’ at first use.

**Response:** We have revised accordingly.

**Revised text in Abstract:**

**Page 3 lines 63-67** We further found that the overall point estimate for the risk of ICC over 10 years of follow-up among women in the primary HPV arm was 0.39 compared to women in the primary cytology arm, but this was not statistically significant (IRR: 0.39; 95% confidence interval, CI [0.14, 1.09]; *p*=0.0726).

5) l.67, please change to: “For women over 55 years of age,…”. When presenting age, please add a unit, such as ‘years’. Please revise throughout the manuscript, including tables and figures (including those in the Supporting Information).

**Response:** Thank you for the comment. We have revised throughout our manuscript and supporting information for the expression.

6) ll.67-69: Please rewrite the Abstract conclusion, as we feel that the phrase "substantially more reassuring" refers to the participants' perception, which was not assessed here. Please use more technical language; the phrase "In this study, we observed ..." may be useful.

**Response:** Thank you for the comment. We have revised our language in the conclusion.

**Revised text in Abstract:**

**Page 4 lines 75-78** In this study, we observed that women over 55 years of age who received a primary negative HPV test result had substantially lower risk of CIN2+, and ICC, compared to women who received a primary negative cytology result. This should apply even if the screening interval were prolonged to 7 years.

7) In the last sentence of the Abstract Methods and Findings section, please describe the main limitation(s) of the study's methodology.

**Response:** Thank you for the comment. We have added the main limitation in the end of the methods and findings section.

**Revised text in Abstract:**

**Page 4 lines 71-74** We were limited by a partial imbalance of invitations during the follow-up between the two arms which may have led to an underestimation of the effectiveness of primary HPV-based screening.

AUTHOR SUMMARY

1) Please revise the formatting; each sub-heading should contain 2-3 single sentence, concise bullet points.
**Response:** Thank you for the comment. We have revised our language to make the author summary more concise

**Revised text in Author Summary:**

**Page 5-6 lines 104-144**

Why Was This Study Done?

Cervical screening in women above the age of 55-60 years is challenging, and invasive cancer still occurs in this age group.

Controversies remain on the choice of optimal test, the interval, the upper age limit and criteria for discontinuing screening.

What Did the Researchers Do and Find?

We performed a long-term follow-up of a randomized healthcare policy trial including 14719 women in Stockholm-Gotland region of Sweden, comparing the effectiveness of primary HPV-based screening and primary cytology-based screening among women aged 56-61 years.

Women who tested negative by primary cytology at the age of 56-61 still had a noticeable risk of cervical cancer over the study period, whereas women who tested negative by primary HPV had an 84% lower risk of cancer.

Primary HPV testing had higher longitudinal sensitivity in detecting precancerous lesion at 5 years, 7 years, and even at 10 years, respectively, after the baseline test compared to primary cytology testing.

What Do These Findings Mean?

For women older than 55-60, those who receive a negative HPV result are at substantially lower risk of cervical cancer compared to those who receive a negative cytology result.

The screening interval could conceivably be extended to 7-10 years, when an HPV-based cervical screening policy is adopted.

We conclude that for women to exit cervical screening at the age of around 60 with only a primary cytology negative result is not optimal.

We may have slightly underestimated the overall performance of primary HPV-based screening due to women in cytology arm ultimately having undergone a more intensive screening.

2) In the final bullet point of 'What Do These Findings Mean?', please include the main limitations of the study in non-technical language.

**Response:** Thank you for the comment. We have added the main limitation of the study in the end.

**Revised text in Author Summary:**

**Page 6 lines 143-144** We may have slightly underestimated the overall performance of primary HPV-based screening due to women in cytology arm ultimately having undergone a more intensive screening.

INTRODUCTION
1) l.95: Please define ‘IR’ at first use.

**Response:** Thank you for the comment. We have revised accordingly.

**Revised text in Introduction:**

**Page 7 lines 146-148** We may have slightly underestimated the overall performance of primary HPV-based screening due to women in cytology arm ultimately having undergone a more intensive screening.

2) l.106: Please define ‘ICC’ at first use.

**Response:** Thank you for the comment. We have revised accordingly.

**Revised text in Introduction:**

**Page 7 lines 156-159** An efficient cervical cancer screening program can provide early detection of precancerous lesions which can lead to early treatment intervention and eventually reduction in risk of invasive cervical cancer (ICC); as well as providing early detection of manifest ICC.

METHODS AND RESULTS

1) l.190: Please define ‘AIS’ at first use.

**Response:** Thank you for the comment. We have revised accordingly.

**Revised text in Method:**

**Page 11 lines 248-253** We identified CIN2+ (includes CIN2, CIN3, Adenocarcinoma in situ, ICC) diagnosed by a histopathology test in the NKCx. In the analysis of histopathology-confirmed CIN2+ as outcome, the follow-up time was defined as the time elapsed from the baseline test to either the first histopathological diagnosis of CIN2+, or the date of the last registered test, including cytology, HPV, and histopathology before 31^st^ Dec 2022.

2) Figure 1/2/3: Please define ‘HPV’ in the figure description.

**Response:** Thank you for the comment. We have revised accordingly.

**Revised text in Results:**

**Page 15 lines 345-348** Fig 1. Flow chart of the study population. HPV, human papillomavirus; CIN1+, cervical intraepithelial neoplasia grade 1 or worse; CIN2+, cervical intraepithelial neoplasia grade 2 or worse; ICC, invasive cervical cancer; all the lesions were diagnosed by histo-pathological test; end of follow-up: 2022-12-31.

**Page 17 lines 364-374** Fig 2. Cumulative incidence of CIN2+ and ICC over 10 years by test methods and baseline results. HPV, human papillomavirus; CIN2+, cervical intraepithelial neoplasia grade 2 or worse; ICC, invasive cervical cancer; follow-up time for CIN2+ was defined as the date of baseline test to the date of the last registered test. Death, emigration and total hysterectomy were considered as competing events for outcome ICC; * artificial increase due to censoring.

Fig 3. Cumulative incidence of CIN2+ and ICC over 10 years by baseline results. HPV, human papillomavirus; CIN2+, cervical intraepithelial neoplasia grade 2 or worse; ICC, invasive cervical cancer; follow-up time for CIN2+ was defined as the date of baseline test to the date of the last registered test. Death, emigration and total hysterectomy were considered as competing events for outcome ICC; * artificial increase due to censoring.

3) Table 1: Please define ‘HPV’, ‘Q1-Q3’, and ‘ICC’ below the table.

**Response:** Thank you for the comment. We have revised accordingly.

4) Figure 3: Please note that in Figure 3 the explanation for the asterisk is missing.

**Response:** Thank you for the comment. We have added the explanation in the figure and the caption.

5) Table 2/3/4: Please define ‘HPV’ below the table.

**Response:** Thank you for the comment. We have revised accordingly.

6) l.318: Please report statistical information as follows to improve clarity for the reader "22% (95% CI [13%,28%]; p</=). For example: “(IRR: 0.39, 95% CI [0.14,1.09], p=0.0726, Table 4)” When reporting 95% CIs please separate upper and lower bounds with commas instead of hyphens as the latter can be confused with reporting of negative values. Please revise throughout the manuscript.

**Response:** Thank you for the comment. We have revised throughout the manuscript accordingly.

7) ll.319-321, please change to: “Among women with baseline negative results, women with a primary HPV negative result had significantly lower IRs of CIN2+ (IRR: 0.32, 95% CI [0.18,0.55], p<0.0001) and ICC (IRR: 0.16, 95% CI [0.04,0.72], p=0.0163, Table 4).”

**Response:** Thank you for the comment. We have revised accordingly.

**Revised text in Results:**

**Page 22 lines 399-402** Among women with baseline negative results, women with a primary HPV negative result had significantly lower risks of CIN2+ (IRR: 0.32, 95% CI [0.18, 0.55], *p<*0.0001, Table 4) and ICC (IRR: 0.16, 95% CI [0.04, 0.72], *p*=0.0163, Table 4).

8) l.316ff: Please check carefully that you have used 'IR' or 'IRR' appropriately in the text. We have noticed that you have used the term 'IR' when presenting 'IRR' values in parentheses. Please revise throughout the entire main text.

**Response:** Thank you for the comment. We have carefully checked our manuscript to avoid this mistake. We also replaced some IR with risk to avoid confusion.

9) ll.332-339: Please revise according to comment 7 (i.e. two separate parentheses).

**Response:** Thank you for the comment. We have revised accordingly.

**Revised text in Results:**

**Page 22 lines 413-416** Overall, women with a previous abnormality had higher risk of CIN2+ (IRR: 2.5, 95% CI [1.6, 3.9], *p*<0.0001) and similar risk of ICC (IRR: 1.4, 95% CI [0.3, 6.1], *p*=0.6503) compared to women without a previous abnormality.

DISCUSSION

1) l.384: Please remove the word ‘respectively’.

**Response:** Thank you for the comment. We have revised accordingly.

**Revised text in Discussion:**

**Page 25 lines 533-534** To our knowledge, we are the first study to report the incidence rate of ICC in this age group after a primary HPV positive and cytology positive test.

2) l.415, please change to "HPV-based screening”.

**Response:** Thank you for the comment. We have revised accordingly.

**Revised text in Discussion:**

**Page 26 lines 564-567** Our data support that primary HPV-based screening is more reliable in protecting against ICC than primary cytology-based screening and older women may benefit more from changing the primary test from cytology to HPV.

3) l.486: Please remove the conclusion subheading. The Conclusion paragraph should be a continuous part of the Discussion section.

**Response:** Thank you for the comment. We have removed the conclusion subheading accordingly.

REFERENCES

For references 3 and 25, please remove one of the dates. We only need you to include the access date (e.g., [accessed: 10/04/2024]).

**Response:** Thank you for the comment. We have revised accordingly.

**Revised text in Reference:**

**Page 32 reference 3:** Global strategy to accelerate the elimination of cervical cancer as a public health problem [Internet]. Available from: <https://www.who.int/publications-detail-redirect/9789240014107> [accessed: 12/08/2024]

SUPPLEMENTARY MATERIAL

1) Please ensure to reference all supporting material in the main text.

**Response:** Thank you for the comment. We have checked that all supporting material is referred in the main text.

2) Please note that in the S4 Support Information you have misspelled the heading "Screening Protocol".

**Response:** Thank you for the comment. We have revised accordingly.

3) In the published article, supporting information files are accessed only through a hyperlink attached to the captions. For this reason, you must list captions at the end of your manuscript file. You may include a caption within the supporting information file itself, as long as that caption is also provided in the manuscript file. Do not submit a separate caption file.

**Response:** Thank you for the comment. The caption of the support information has been placed right after the conclusion in the manuscript.

**Revised text in Supporting information:**

**Page 30 lines 633-670:**

Supporting information

**S1 CONSORT Checklist.** CONSORT, Consolidated Standards of Reporting Trials. (DOCX)

**S2 Study Protocol.** Randomized Implementation of Primary HPV Testing in the Organized Screening for Cervical Cancer in Stockholm. HPV, human papillomavirus. (PDF)

**S3 Fig.** Process flowchart of cervical screening for women in this trial. CIN2+, cervical intraepithelial neoplasia grade 2 or worse

**S4 Supporting Information.** Supplementary protocol, statistical analysis and tables. (DOXC)

**S5 Statistical Analysis Plan.** (DOXC)

When SI files are contained with a single file:
Please label the file as ‘S1 Supporting Information’.
Please apply alphabetical labelling to each table and figure contained within the S1 file. For example, ‘Fig A’ to ‘Fig Z’ and ‘Table A’ to ‘Table Z’.
Plain text does not need to be labelled and can just be given a title as necessary. For example, ‘Statistical Analysis Plan’.
Please cite tables/figures as ‘Fig A in S1 Supporting Information’ and/or ‘Table A in S1 Supporting Information’, for example.
Please cite plain text as, ‘Statistical Analysis Plan in S1 Supporting Information’, for example.

**Response:** Thank you for the comment. We have revised accordingly.

When SI files are uploaded as separate files:
Please label tables as ‘S1 Table’ (so on) and figures as ‘S1 Fig’ (and so on).
Any additional documents (protocols/analysis plans etc.) can be labelled as ‘S1 Protocol’, for example. Please cite items as exactly as labelled.

**Response:** Thank you for the comment. We have revised accordingly.


SOCIAL MEDIA

To help us extend the reach of your research, please provide any X (formerly known as Twitter) handle(s) that would be appropriate to tag, including your own, your co-authors’, your institution, funder, or lab. Please enter in the submission form any handles you wish to be included when we post about this paper.

**Response:** Thank you for offering to help extend the reach of our research. We currently do not have any X (formerly known as Twitter) handles to provide. However, we would appreciate the opportunity to improve the visibility of our research through LinkedIn. Please feel free to tag Joakim Dillner and Karin Sundström from Karolinska Institutet on LinkedIn in any posts about this paper and include the topic #CervicalCancer.

------------------------------------------------------------
General Editorial Requests

1) We ask every co-author listed on the manuscript to fill in a contributing author statement. If any of the co-authors have not filled in the statement, we will remind them to do so when the paper is revised. If all statements are not completed in a timely fashion this could hold up the re-review process. Should there be a problem getting one of your co-authors to fill in a statement we will be in contact. YOU MUST NOT ADD OR REMOVE AUTHORS UNLESS YOU HAVE ALERTED THE EDITOR HANDLING THE MANUSCRIPT TO THE CHANGE AND THEY SPECIFICALLY HAVE AGREED TO IT.

**Response:** Thank you for the comment. All authors have finished the author statement. We haven’t added or removed any authors. We have added the clinical affiliation of two authors.

**Revised text in Title Page:**

**Page 1 lines 4-10:**

Qingyun Yao^1*^, Jiangrong Wang^1^, K. Miriam Elfström^1^, Björn Strander^2^, Joakim Dillner^1,3^, Karin Sundström^1,3^

^1^ Center for Cervical Cancer Elimination, Department of Clinical Science, Intervention and Technology, Karolinska Institutet, Sweden

^2^ Institute of Clinical Sciences, Sahlgrenska Academy, University of Gothenburg, Sweden

^3^ Medical Diagnostics Karolinska, Stockholm, Sweden

2) Please ensure that the paper adheres to the PLOS Data Availability Policy (see [https://eur01.safelinks.protection.outlook.com/?url=http%3A%2F%2Fjournals.plos.org%2Fplosmedicine%2Fs%2Fdata-availability&data=05%7C02%7Cqingyun.yao%40ki.se%7Cf3fb3dadb56e4dfea03608dd07cccad3%7Cbff7eef1cf4b4f32be3da1dda043c05d%7C0%7C0%7C638675298372348319%7CUnknown%7CTWFpbGZsb3d8eyJFbXB0eU1hcGkiOnRydWUsIlYiOiIwLjAuMDAwMCIsIlAiOiJXaW4zMiIsIkFOIjoiTWFpbCIsIldUIjoyfQ%3D%3D%7C0%7C%7C%7C&sdata=uheUdccejfAJC79STVOu64kOHf%2FgJxLtfim3WDidfR4%3D&reserved=0)](https://eur01.safelinks.protection.outlook.com/?url=http%3A%2F%2Fjournals.plos.org%2Fplosmedicine%2Fs%2Fdata-availability&data=05%7C02%7Cqingyun.yao%40ki.se%7Cf3fb3dadb56e4dfea03608dd07cccad3%7Cbff7eef1cf4b4f32be3da1dda043c05d%7C0%7C0%7C638675298372348319%7CUnknown%7CTWFpbGZsb3d8eyJFbXB0eU1hcGkiOnRydWUsIlYiOiIwLjAuMDAwMCIsIlAiOiJXaW4zMiIsIkFOIjoiTWFpbCIsIldUIjoyfQ%3D%3D%7C0%7C%7C%7C&sdata=uheUdccejfAJC79STVOu64kOHf%2FgJxLtfim3WDidfR4%3D&reserved=0)" \o "https://eur01.safelinks.protection.outlook.com/?url=http%3A%2F%2Fjournals.plos.org%2Fplosmedicine%2Fs%2Fdata-availability&data=05%7C02%7Cqingyun.yao%40ki.se%7Cf3fb3dadb56e4dfea03608dd07cccad3%7Cbff7eef1cf4b4f32be3da1dda043c05d%7C0%7C0%7C638675298372348319%7CUnknown%7CTWFpbGZsb3d8eyJFbXB0eU1hcGkiOnRydWUsIlYiOiIwLjAuMDAwMCIsIlAiOiJXaW4zMiIsIkFOIjoiTWFpbCIsIldUIjoyfQ%3D%3D%7C0%7C%7C%7C&sdata=uheUdccejfAJC79STVOu64kOHf%2FgJxLtfim3WDidfR4%3D&reserved=0)), which requires that all data underlying the study's findings be provided in a repository or as Supporting Information. For data residing with a third party, authors are required to provide instructions with contact information for obtaining the data. PLOS journals do not allow statements supported by "data not shown" or "unpublished results." For such statements, authors must provide supporting data or cite public sources that include it.

**Response:** Thank you for the comment. We have added information on obtaining the data in the data availability statement.

**Revised text in Data availability:**

**Page 30 lines 672-677** Our data contains potentially identifying patient information, thus according to Swedish law the authors are not able to make the dataset publicly available. The data is deposited with the Swedish Cervical Screening Registry (www.nkcx.se) and can be requested at info@nkcx.se (administrator Sara Nordqvist Kleppe). One can vist http://www.nkcx.se/research_e.htm for detailed information about how to apply for access. The code used in the analysis is available at request from Qingyun Yao (qingyun.yao@ki.se).

3) Please review your reference list to ensure that it is complete and correct. If you have cited papers that have been retracted, please include the rationale for doing so in the manuscript text, or remove these references and replace them with relevant current references. Any changes to the reference list should be mentioned in the rebuttal letter that accompanies your revised manuscript.
**Response:** Thank you for the comment. We have checked all the references. None of them have been retracted. We have changed one citation from the preprint version to the official published version.

**Revised text in Reference:**

**Page 33 reference 25:**

Wang J, Elfström KM, Dillner J. Human papillomavirus-based cervical screening and long-term cervical cancer risk: a randomised health-care policy trial in Sweden. Lancet Public Health. 2024 Nov;9(11):e886–95.
